# Crystal Structure of Glycoprotein C from a Hantavirus in the Post-fusion Conformation

**DOI:** 10.1371/journal.ppat.1005948

**Published:** 2016-10-26

**Authors:** Shmuel Willensky, Hagit Bar-Rogovsky, Eduardo A. Bignon, Nicole D. Tischler, Yorgo Modis, Moshe Dessau

**Affiliations:** 1 The Faculty of Medicine in the Galilee, Bar-Ilan University, Safed, Israel; 2 Dept. of Medicine, University of Cambridge, MRC Laboratory of Molecular Biology, Cambridge, United Kingdom; 3 Molecular Virology Laboratory, Fundación Ciencia & Vida, Santiago, Chile; Washington University, UNITED STATES

## Abstract

Hantaviruses are important emerging human pathogens and are the causative agents of serious diseases in humans with high mortality rates. Like other members in the *Bunyaviridae* family their M segment encodes two glycoproteins, G_N_ and G_C_, which are responsible for the early events of infection. Hantaviruses deliver their tripartite genome into the cytoplasm by fusion of the viral and endosomal membranes in response to the reduced pH of the endosome. Unlike phleboviruses (e.g. Rift valley fever virus), that have an icosahedral glycoprotein envelope, hantaviruses display a pleomorphic virion morphology as G_N_ and G_C_ assemble into spikes with apparent four-fold symmetry organized in a grid-like pattern on the viral membrane. Here we present the crystal structure of glycoprotein C (G_C_) from Puumala virus (PUUV), a representative member of the *Hantavirus* genus. The crystal structure shows G_C_ as the membrane fusion effector of PUUV and it presents a class II membrane fusion protein fold. Furthermore, G_C_ was crystallized in its post-fusion trimeric conformation that until now had been observed only in *Flavi-* and *Togaviridae* family members. The PUUV G_C_ structure together with our functional data provides intriguing evolutionary and mechanistic insights into class II membrane fusion proteins and reveals new targets for membrane fusion inhibitors against these important pathogens.

## Author Summary

Hantaviruses (family: *Bunyaviridae*) encompass pathogens responsible to serious human diseases and economic burden worldwide. Following endocytosis, these enveloped RNA viruses are directed to an endosomal compartment where a sequence of pH-dependent conformational changes of the viral envelope glycoproteins mediates the fusion between the viral and endosomal membranes. The lack of high-resolution structural information for the entry of hantaviruses impair our ability to rationalize new treatments and prevention strategies. We determined the three-dimensional structure of a glycoprotein C from Puumala virus (PUUV) using X-ray crystallography. The two structures (at pH 6.0 and 8.0) were determined to 1.8 Å and 2.3 Å resolutions, respectively. Both structures reveal a class II membrane fusion protein in its post-fusion trimeric conformation with novel structural features in the trimer assembly and stabilization. Our structures suggest that neutralizing antibodies against G_C_ target its conformational changes as inhibition mechanism and highlight new molecular targets for hantavirus-specific membrane fusion inhibitors. Furthermore, combined with the available structures of other class II proteins, we remodeled the evolutionary relationships between virus families encompassing these proteins.

## Introduction

The *Bunyaviridae* is a large and diverse virus family of human, animal and plant pathogens that encompasses five genera; *Phlebovirus*, *Orthobunyavirus*, *Hantavirus*, *Nairovirus* and *Tospovirus*. Members of the *Hantavirus* genus are rodent-borne zoonotic viruses and are important human pathogens responsible for severe illnesses such as hemorrhagic fever with renal syndrome (HFRS), and hantavirus pulmonary syndrome (HPS) [1–4]. Puumala virus (PUUV), the causative agent of a mild form of HFRS was first isolated in Finland [5]. In humans, PUUV infection is mostly asymptomatic or manifested with minor symptoms. However, outbreaks were recently reported in central Europe with growing numbers of affected patients [6–8]. The bank vole (*Myodes glareolus*) is the main reservoir of the virus and transmission to humans occurs typically via aerosols of the rodent excreta with no role for arthropod vectors.

Hantaviruses encompass a tripartite, negative sense ssRNA genome. The viral medium (M) segment encodes the two glycoproteins, G_N_ and G_C_, originating from a glycoprotein precursor (GPC) that is cleaved into N- and C-terminal fragments [9–11]. G_N_ and G_C_ assemble into a lipid bilayer envelope to form an outer protein shell. The non-continuous, pleomorphic envelope projects G_N_ and G_C_ as a spike complex bearing an apparent four-fold symmetry [12]. Recently, the atomic resolution structure of G_N_ was published and together with electron cryo-tomography data it was proposed to be located at the membrane distal part of the spike complex [13]. However the structure, orientation and stoichiometry of G_C_ within the spikes remain unclear.

To deliver their RNA genome into the host cell cytoplasm, hantaviruses must fuse their envelope with a cellular membrane. Like other enveloped viruses, hantaviruses rely on their glycoproteins to induce membrane fusion [14]. Following attachment to the host cell, hantaviruses usually undergo clathrin-mediated endocytosis (CME). Interestingly, clathrin-independent endocytosis was reported for some hantaviruses [15, 16], implying that different routes may be involved in these viruses entry. In both routes, however, the virus is directed to an endosomal compartment where the glycoproteins respond to the reduced pH of the compartment with a sequence of conformational changes [17]. These conformational changes expose a hydrophobic motif, which is inserted into the endosomal membrane [18, 19]. The glycoprotein then folds back on itself, forcing the cell membrane (held by the fusion motif) and the viral membrane (held by a transmembrane anchor) to proximity, inducing the viral and endosomal membranes to fuse [20–22].

Based on bioinformatic studies and *in vitro* experiments using synthetic peptides it was postulated that hantavirus G_C_ adopts a class II membrane fusion protein fold [23, 24]. Until recently, viral class II fusion proteins were thought to be restricted to members of the *Flavivirus* genus (family: *Flaviviridae*) and the *Togaviridae*. However, the crystal structure of G_C_ from Rift Valley fever virus (RVFV—family *Bunyaviridae*, genus: *Phlebovirus*) showed that the class II fold extends beyond these two families [25]. Interestingly, not all *Flaviviridae* members contain a class II membrane fusion protein as bovine viral diarrhea virus (BVDV, genus: Pestivirus) E2 protein and hepatitis C virus E2 (HCV, genus: Hepacivirus) exhibit completely different folds in their proposed fusion proteins [26, 27]. In the absence of high-resolution structures for the complete E1 proteins from these viruses this data suggests that BVDV and HCV (flavivirus) fusion proteins do not adopt a class II fold.

The transition of class II membrane fusion proteins from their pre-fusion homo- or heterodimers on the virus surface to a post-fusion homotrimer has been shown to depend on the acidification of the virus’ environment [21, 28–30]. Recently, Acuña and colleagues have shown that G_C_ from Andes virus (ANDV, genus: *Hantavirus*) forms trimers in response to acidic environment at pH 5.5 [17]. Hantavirus fusion activity was also demonstrated by syncytia formation upon low pH treatment of Vero E6 cells expressing G_N_ and G_C_ glycoproteins [14, 31]. In this cellular context, a pH of 5.9 was found to activate fusion of Andes virus while a pH of 6.3 was reported as the activation threshold for Hantaan virus [14, 32].

In the absence of experimental high-resolution structural data for G_C_, the molecular basis of membrane fusion in hantaviruses remains obscure. Here we present the first high-resolution structure of a fusogen from the hantavirus genus.

## Results and Discussion

### PUUV G_C_ is a class II membrane fusion protein

The ectodomain of PUUV G_C_ spans residues 659–1114 (GPC numbering, 1–456 in G_C_ numbering). To obtain soluble protein for structural studies, we expressed only PUUV G_C_ residues 659–1106 (1–448, soluble G_C_ or sG_C_) using baculovirus expression system and purified it to homogeneity (see material and methods). During the elution step of ion exchange (IEX) chromatography we obtained two populations (termed sG_C_^XF1^ and sG_C_^XF2^) that each crystallized in a distinct crystal form. We then determined the crystal structures of sG_C_^XF1^ and sG_C_^XF2^ to 1.8 Å and 2.5 Å resolution, respectively, with excellent crystallographic statistics (Table 1). Although sG_C_^XF1^ crystals appeared in pH 6.0 and sG_C_^XF2^ in pH 8.0, in both crystal forms PUUV sG_C_ adopts the three-domain architecture of the post-fusion conformation of class II viral fusion proteins. It is not unprecedented that some class II membrane fusion proteins were crystallized in their post-fusion conformation without low pH triggering [33, 34], however we cannot exclude that for sG_C_^XF2^ the pH was not changed during the crystallization period. The overall structure in both crystal forms is similar so to simplify our discussion we will refer mainly to the sG_C_^XF1^ unless mentioned otherwise.

**Table 1:** Crystallographic data collection and refinement statistics.

	sG_C_^XF1^				sG_C_^XF2^
	SIRAS^native^	SIRAS^Hg^	Native		Native
**Data collection**					
Space group	R 3 2: H	R 3 2: H	R 3 2: H		I 2_1_ 3
Cell dimensions					
*a*, *b*, *c* (Å)	96.4, 96.4, 246.3	96.4, 96.4, 246.2	96.4, 96.4, 247.1		138.5, 138.5, 138.5
*α*, *β*, *γ* (°)	90, 90, 120	90, 90, 120	90, 90, 120		90, 90, 90
Wavelength (Å)	1.8	1.009	0.97949		0.97949
Resolution (Å)^a^	50–2.5 (2.6–2.5)	50–2.7 (2.8–2.7)	48–1.8 (1.9–1.8)		49–2.5 (2.53–2.5)
Unique reflections	29,580 (3304)	23,488 (2416)	41,365 (6027)		15391 (525)
*R*_merge_ (%)^b^	14.9 (114.7)	11.9 (144.2)	9.4 (138)		7.5 (78.3)
*I*/*σI* ^a^	15.8 (2.5)	15 (1.03)	14.65 (1.38)		14.43 (2.15)
Completeness (%)^a^	100 (100)	99.9 (99.5)	99.9 (99.9)		99.6 (99.6)
Redundancy^a^	14.83 (14.66)	9.8 (5.33)	9.93 (9.01)		5.46 (5.5)
Overall figure of merit		0.49			
**Refinement**					
Resolution (Å)			48.2–1.8		49–2.5
No. reflections			41,357		14,611
*R*_work_ / *R*_free_ ^c^			18.3/20.9		20.8/25.8
Averaged B factor (Å^2^)^e^			38.48 / 3534		63.63/3238
macromolecules^e^			37.62/ 3143		63.23/3149
ligands^e^			65.31/ 70		106.6/44
solvent^e^			41.02/ 321		49.78/45
**R.M.S deviations** ^**d**^					
Bond lengths (Å)			0.009		0.008
Bond angles (°)			0.893		1.271
**Ramachandran analysis**					
In preferred regions (%)				97.1	94
In allowed regions (%)				2.7	5.3
Outliers (%)				0.2	0.7
**Synchrotron Beamline**	BESSY 14.1	ESRF ID30B		DLS I04	DLS I04

^a^ Highest resolution shell is shown in parentheses.

^b^ *R*_merge_ = *Σ*_*hkl*_ *Σ*_*i*_ *|I*_*hkl*,*i*_ *–< I>*_*hkl*_ *| / Σ*_*hkl*_ *Σ*_*i*_ *|I*_*hkl*,*i*_ *|*, where I_hkl_ is the intensity of a reflection and *<I>*_*hkl*_ is

the average of all observations of the reflection.

^c^ *R*_*free*_, *R*_*work*_ with 10% of F_obs_ sequestered before refinement.

^d^ R.M.S., root mean square.

^e^ The number on the right is the number of atoms that the b-factor was calculated for.

**Fig 1:**
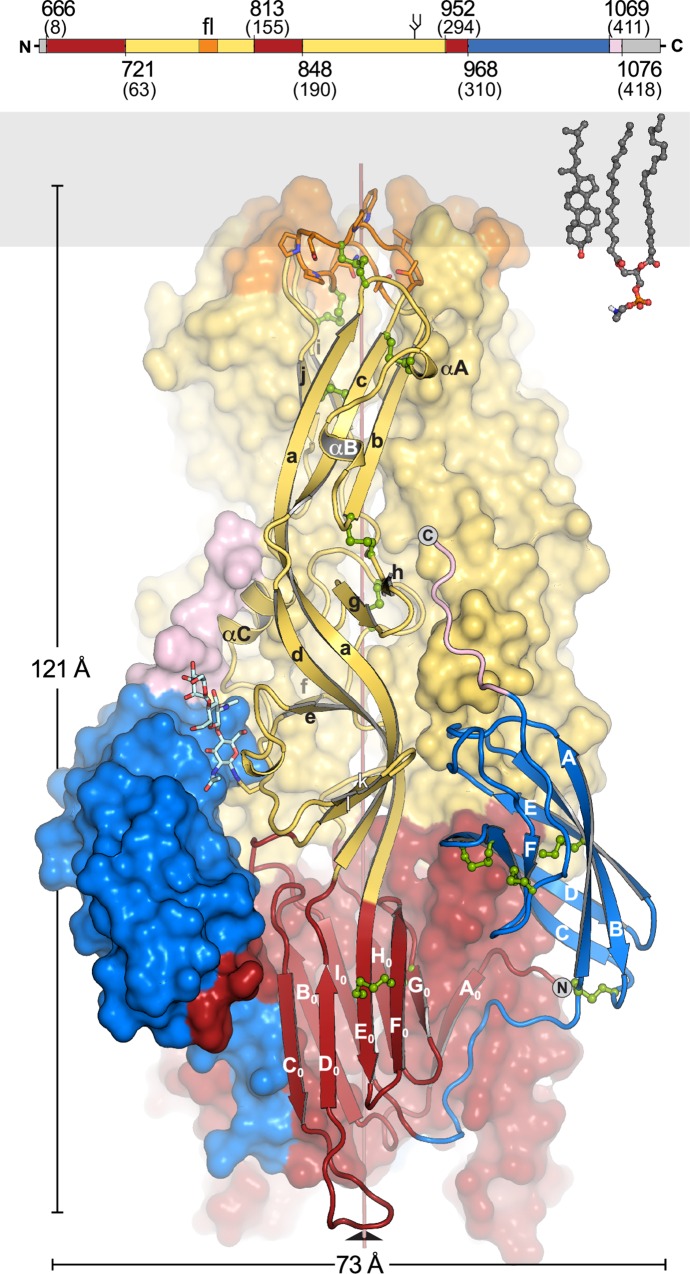
Overall fold of the post-fusion PUUV sG_C_. PUUV sG_C_ has the same three-domain architecture as other class II proteins. Domain I is shown in red, domain II in yellow with the fusion loop in orange, domain III in blue and the stem region in light pink. Residue numbers follow GPC numbering. The membrane proximal part of the stem, the transmembrane anchor and the cytoplasmic tail (grey) are missing in the structure. Secondary structure elements are indicated. Glycans are linked to N937. Disulfide bonds are in green. Gray rectangle represents the outer leaflet of the membrane. On the right, a cholesterol and phosphatidylethanolamine molecules are shown for scale. On the top is linear domain organization of PUUV G_C_. Color scheme is as described for the structure. Gray indicates regions that were not observed in the structure. Numbers correspond to GPC numbering and in parenthesis is G_C_ numbering.

Viral class II membrane fusion proteins were found previously only in flaviviruses, alphaviruses, rubivirus and more recently in a phlebovirus [25, 35–37] (Figs 1, S1). The crystal structure of PUUV sG_C_ spans residues 666–1076 (GPC numbering), lacking seven N-terminal and 30 C-terminal residues of the expressed ectodomain. Domain I, an eight-stranded β-sandwich (with strands termed B_0_-I_0_), is the center of the structure that arranges domain II and III around it (Fig 1). Two insertions in domain I between strands D_0_-E_0_ and strands H_0_-I_0_ form the elongated, mostly β-stranded domain II. The putative fusion loop, the endosomal membrane anchor, is located on the part of domain II that is distal to domain I. Domain III is an IgC-like module with six β-strands and is followed by a segment of eight amino acids of the so-called stem region.

Compared to fusion proteins from flaviviruses and alphaviruses, PUUV G_C_ has a longer stem region connecting domain III to the transmembrane (TM) domain. The stem region of PUUV G_C_ spans approximately 44 residues, including two conserved cysteines (S2 Fig). Due to its disordered nature we could not detect electron density for most of this region. However, the first eight residues of the stem (1068–1076) could be modeled in both, sG_C_^XF1^ and sG_C_^XF2^. The last residue visible in both of our structures is T1076, which lays ~30 Å from the fusion loop (Fig 1). The remaining 38 residues connecting to the TM anchor can easily cover the distance to the fusion loop. The overall domain organization (in particular the position of domain III), the parallel trimeric assembly and the stem peptide directionality imply that our structure represents sG_C_ in its post-fusion conformation, or at least in the final stages of the fusion between the viral and the host-cell membranes.

PUUV sG_C_ from both preparations (sG_C_^XF1^ and sG_C_^XF2^) is a monomer in solution as determined by size exclusion chromatography (SEC) (S3A Fig). To investigate the oligomeric state of sG_C_ at different pHs, we used size exclusion chromatography combined with multiangle light scattering (SEC-MALS) at pH 8.0 and pH 5.0. Unexpectedly, we found that low pH does not trigger sG_C_ trimerization in solution as sG_C_ scatters as a monomer even at pH 5.0 (S3B Fig). Elution of sG_C_ was significantly retarded at pH 5.0 compared to pH 8.0, most likely due to non-specific interaction of the protein with the dextran resin [38]. The same effect was reported also for RVFV G_C_ ectodomain [25]. Nevertheless, in both PUUV sGc structures, one molecule in the asymmetric unit assembles into a homotrimer around the crystallographic three-fold axis (Fig 1). The protomers adopt the post-fusion domain arrangement, resembling other class II post-fusion structures [14, 20, 21, 33, 34] (S1 Fig). They associate in a parallel arrangement with the fusion loop placed at the same end of a stable elongated molecule. The C-terminal stem region is pointing towards the target membrane (Fig 1).

PUUV G_C_ and RVFV G_C_ (Genus: *Phlebovirus*), both members of the *Bunyaviridae* family, share some structural features that are different from other class II proteins. Similar to phleboviruses, G_C_ from hantaviruses has a high cysteine content, with 26 cysteine residues. In our structure we located 24 cysteines involved in 12 disulfide bonds. Electron density for the remaining cysteines (C1094 and C1098), at the C-terminal end of the protein, could not be detected. It was suggested before that the ^787^C-X-X-C^790^ motif, mapped to domain II, might be involved in disulfide rearrangement to prevent hantavirus inactivation under conditions of low-pH treatment [39]. In our structure, C787 and C790 are located at the membrane proximal region of domain II and are involved in two different disulfide bonds (with C749 and C913, respectively). From the only other *Bunyaviridae* fusogen structures (RVFV G_C_ in its pre-fusion and pre-hairpin conformations, PDB ID 4HJ1 and 4HJC, respectively), the analogous cysteines have a similar arrangement [25] despite the hinge motions between the two conformations. Therefore, from comparing these two structures with the post-fusion structure of PUUV sG_C_ we conclude that in contrast to the fusogen activation in some class I membrane fusion proteins, where disulfide rearrangement is essential for preventing a premature fusion [40], these disulfides do not reorganize. Instead, they are responsible to rigidify the structure and stabilize the orientation of the putative fusion loop.

### The putative fusion loop of PUUV sG_C_ displays canonical features of a class II endosomal membrane anchor

**Fig 2:**
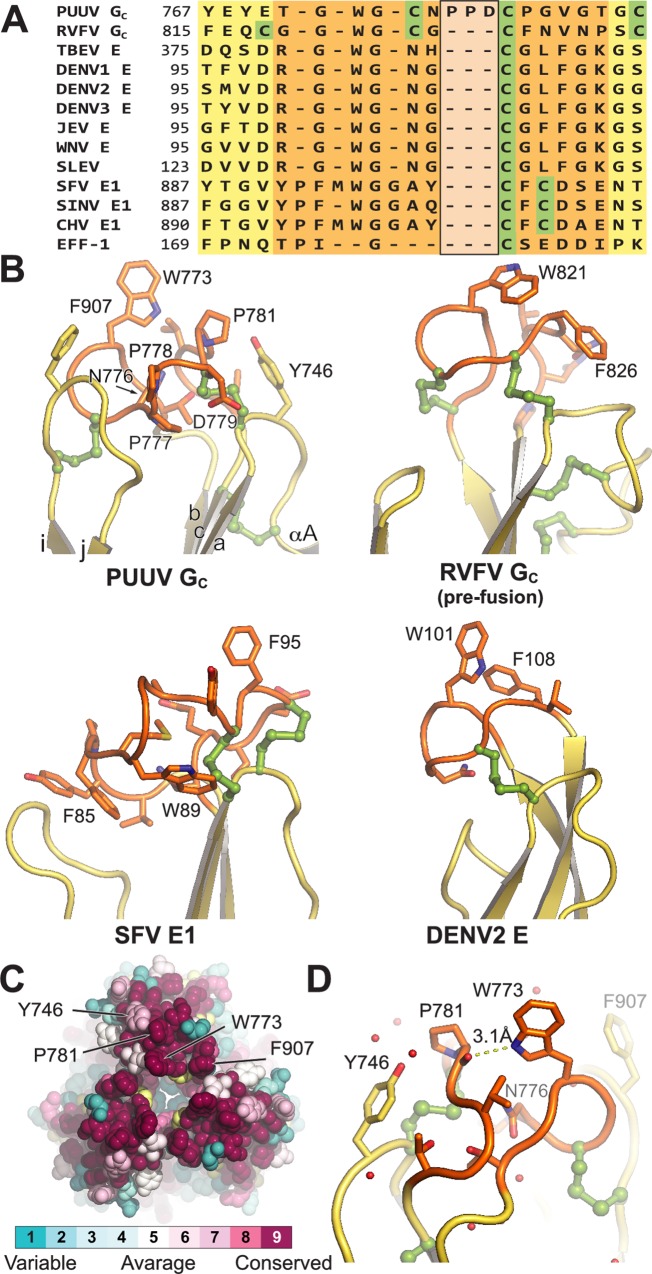
The putative fusion loop of PUUV G_C_ compared to fusion loops of other viral class II membrane fusion proteins. (A) Structure-based multiple sequence alignment of the fusion loop regions from different class II members. Shading is in the same color scheme as in Fig 1. The PXD insertion in PUUV G_C_ is highlighted with a black box. Residues of PUUV G_C_ and RVFV G_C_ correspond to polyprotein precursor numbering. (B) Clockwise from the top left: PUUV G_C_, RVFV G_C_, DENV2 E and Semliki forest virus (SFV) E1 fusion loops (PDB codes 5J81, 4HJ1, 1OK8 and 1RER, respectively). The hydrophobic residues that anchor the protein to the cellular membrane are shown in stick representation and the disulfide bonds are shown in ball-and-stick representation. (C) CONSURF analysis [74] of unique hantavirus G_C_ sequences projected on the surface of PUUV sG_C_ crystal structure. A top view on the fusion loop, down the crystallographic tree-fold axis. The following uniprot (http://www.uniprot.org/) entries were used for the analysis: M9QRJ8, Q9QIZ1, M9QSR6, W5RRK8, Q5MYC0, Q9WJ31, Q2V8Y2, A0A068EN08, A0A0A7EQ65, Q66753, M9QY05, A0S5D7, O12371, Q9WSK6, Q806Y7, A0A0D5W3U2, F1T2C3, A8RRS6, G0WJH7, P27315, C7AGW1, B1NSM7, Q83887, A0A075IFP0, A0A0K0K9P4, Q9WMK6, A0A0F6T9U0, H8XZQ0, Q9DXJ5, Q9E158, Q91BQ9, A0A068ETZ4, A6MD75, B6DDK4, V9MFN9, H6WCQ9, Q99BV0, A0A077D3A4, P08668, F6KBJ3, A0RZG8, K4MYY7, R4JAI4, U5L2G2, O55348, H8ZHK6, H8ZHL5 (D) Hydrogen bond between W773 and the carbonyl of P781. Yellow dash line represents the distance between P781 carbonyl and W773.

Our structure provides a direct view on the putative endosomal membrane anchor of G_C_ known as the fusion loop and contained between β strands *c* and *b* (Fig 2). It was previously demonstrated for Andes virus (ANDV) G_C_, a member of the *Hantavirus* genus, that single mutations in the conserved residues W773, N776 and D779 (W115, N118 and D121 in G_C_ numbering) located at the fusion loop eliminate cell-cell fusion activity and ANDV pseudotyped particles infectivity [32]. From our structure it is apparent that W773 and P781 form a conserved hydrophobic surface (Fig 2B and 2C), exposed towards the target membrane. The N-H group of the W773 side chain forms a hydrogen bond with the carbonyl oxygen of P781, reducing its hydrophilicity and thereby favors the penetration of the fusion loop into the outer leaflet of the endosomal membrane (Fig 2D). This interaction was reported also for dengue virus E trimer where W101 is interacting in the same way with the carbonyl group of G106 [21]. Notably, the side chain of the charged D779, also located in the fusion loop, is pointing to the opposite direction, away from the purportedly membrane plane. Notably, the essential residue N776 maintains a network of hydrogen bonds principally with the main chain carbonyls of residues C780, G782 and with the amine group of residue G785. Therefore, N776 stabilizes the architecture of the fusion loop, thus explaining its importance for fusion. The fusion loop of PUUV G_C_ contains other genus-specific features. It has a three-residue insertion (^777^P-X-D^779^) conserved among hantaviruses (Figs 2A, S2) where X is typically a proline but can be replaced by serine or glycine (S2 Fig). Unlike post-fusion trimers from the *Flavivirus*, A*lphavirus* and *Phlebovirus* genera, the hydrophobic surface at the tip of domain II is extended by the conserved F907 positioned at the loop connecting strands *i* and *j* (Fig 2B). Even though it is less conserved, Y746 located at a third loop contained between strand *a* and *αA* helix, might participate in the membrane anchoring as its side chain directing towards the target membrane and is nearly at the same plane of the other hydrophobic side chains of the fusion loop (Fig 2B and 2C).

### Interactions between protomers in the PUUV sG_C_ post-fusion trimer

**Fig 3:**
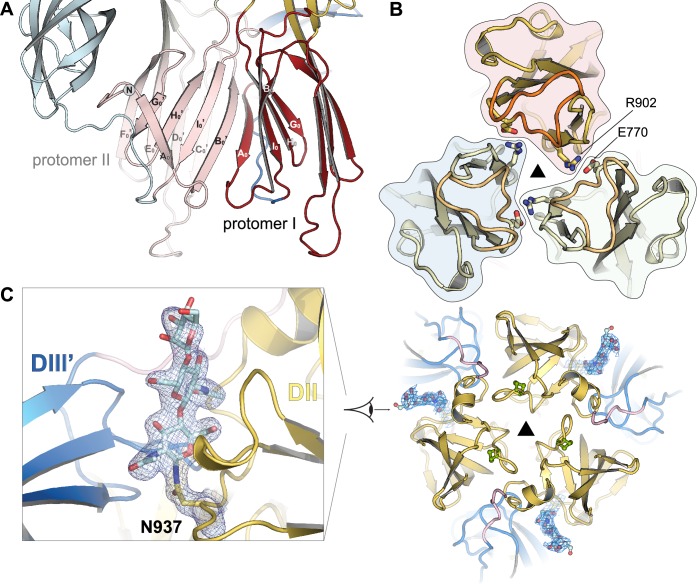
Inter-protomer interactions unique to PUUV G_C_. (A) Strand A_0_ at the N-terminus of domain I extends the B_0_-I_0_-H_0_-G_0_ β-sheet of the adjacent protomer. The donor protomer (protomer 1) is indicated in the same color scheme as in Fig 1 while the neighboring protomer (protomer 2) is shown in faded colors. (B) Inter-trimer salt bridge at the membrane proximal part of domain II. Ionic pairs are in sticks representation. The boundaries of each protomer are highlighted. (C) The glycosylation on N937 mediates interactions between protomers. Right: view of the trimer from the membrane, down the crystallographic three-fold axis. Left: Close-up view on the glycosylation groove between the protomers. N937 and the glycans are in sticks representation. 2F_O_-F_C_ electron density map at 1σ is shown in light blue mesh.

PUUV sG_C_ trimerizes through central interactions in domain I and in the domain-I proximal half of domain II. The total surface buried in trimer interfaces is 5850 Å^2^ (1950 Å^2^ per monomer), 17% larger than in DENV2 E trimer (PDB code 1OK8), but only 3% larger than in the Semliki forest virus (SFV) E1 trimer (PDB code 1RER). In addition to the extensive trimerization interface, there are few elements that are exclusive to the PUUV sG_C_ trimer: unlike other class II members, PUUV G_C_ has an N-terminal extension of domain I that donates a strand, A_0_, to the B_0_-I_0_-H_0_-G_0_ β-sheet from the neighboring protomer, creating an intermolecular continuous beta sheet (Figs 1 and 3A). This N-terminal extension has not been found in structures from the well characterized class II fusion proteins, including that of phlebovirus G_C_ [25], and therefore it seems to be a unique feature of hantaviruses. Cross-protomer interactions are not common in class II trimers. Typically, the protomers are packed against one another making interactions between secondary structure elements in adjacent protomers. A cross-protomer swap was reported only in Rubella virus E1 protein where the C-terminal stem region donates two strands to two different β-sheets of a neighboring protomer [34]. Additionally, there are few cross-protomer salt bridges in the PUUV sG_C_ trimer. The most notable one is at the membrane proximal part of domain II, close to the fusion loop, where E770 forms a salt-bridge with R902 from the neighboring molecule (Fig 3B), thereby stabilizing the trimer in the membrane-proximal region. To functionally test the significance of this salt-bridge in a hantavirus glycoprotein-mediated cell-cell fusion assay (14, 32) we introduced an alanine substitution of R902 (R244 in G_C_ numbering) into PUUV GPC. In addition, the same mutation was also introduced to GPC of ANDV to exploit several approaches that have been established for this virus. The G_C_ sequence of hantaviruses is highly conserved and amounts in the case of PUUV and ANDV to 76% of identity and 89% of similarity (S2 Fig). When cells expressed the wild type and R902A constructs of PUUV and ANDV, G_C_ localized efficiently at the cell surface (S4A Fig). Upon acid-induced incubation, the PUUV and ANDV G_C_ R902A mutants induced syncytia as the wild type proteins (S4B Fig), indicating that the inter-protomer salt bridge may have a less crucial role for fusion activity (see discussion below).

PUUV G_C_ is predicted to have two glycosylation sites, N898 and N937. In our crystal structure we observed N-linked glycans only on N937 whereas N898 is buried in the trimer interface with no available space to accommodate a glycan chain. We therefore conclude that N898 is not glycosylated. In contrast to other class II post-fusion trimers, where the glycans decorate the perimeter of the trimer assembly, in PUUV G_C_ the glycans linked to N937 are tightly packed between domain II of one protomer and domain III of the neighboring protomer (Fig 3C). Except one hydrogen bond between N999 (domain III) and the first N-acetylglucosamine residue, all contacts with the glycans are via hydrophobic interactions. Indeed, it was previously reported that eliminating the glycosylation on N928 in Hantaan virus G_C_ (analogous to PUUV G_C_ N937) is sufficient to prevent cell fusion [41]. Based on our structure and the previous biochemical data, we conclude that the contribution of the glycans to the PUUV G_C_ trimer interface is a key element in stabilizing trimer assembly in hantaviruses.

### Monoclonal neutralizing antibodies against PUUV G_C_ target the membrane fusion mechanism

**Fig 4:**
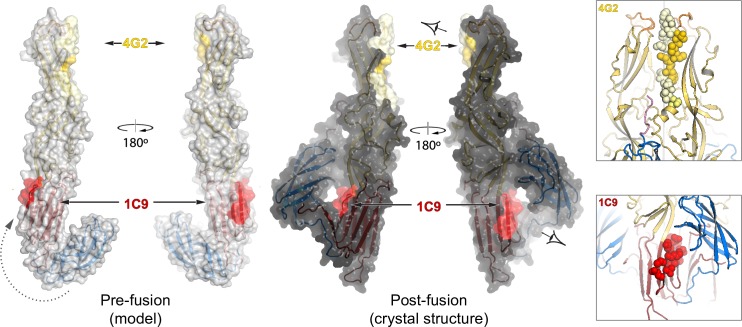
Neutralizing epitope mapping on the surface of PUUV G_C_. Solvent accessible surface representation of the PUUV G_C_ protomer with the linear epitopes of 1C9 (residues 822–834) and 4G2 (residues 903–920) MAb highlighted. Dark-surface protomers are the crystal structure of PUUV G_C_ and bright-surface protomers are pre-fusion model based on PUUV G_C_ domains superimposed onto Semliki forest virus E1 in the pre-fusion conformation (PDB code 2ALA). Gray dashed line represents the movement of domain III between the pre- and post-fusion conformations. On the right, a cartoon representation of the two epitope sites in the context of the trimer. Residues of the linear epitopes are highlighted with sphere representation. View angles are represented by eye symbols.

Previous studies on Hantaan virus (HNTV) neutralizing monoclonal antibodies (MAb) against G_C_ showed sequence dependent reactivity. While the antibodies cross-reacted with other hantaviruses (SEOV, DOBV), they failed to neutralize PUUV [42]. In addition, binding of neutralizing and non-neutralizing MAbs to HNTV G_C_ was mapped to a region that include most of domain III but no specific epitope was determined [43]. Several neutralizing MAb against PUUV have been selected [44–46], two of which were shown to recognize G_C_ (human MAb 1C9 and bank vole MAb 4G2). A peptide scan assay was used to identify the linear epitopes for these MAb [39, 47, 48]. The epitopes for 1C9 and 4G2 MAb map to domain I and II, respectively, and both epitopes contribute to the trimer interface (Fig 4). The G_N_-G_C_ dissociation at pH 6.2–6.4 [39] implies exposure of epitopes in G_C_ that were previously buried or partially exposed in the assembled virion. However it seems that each antibody targets a different stage in the membrane fusion process. In class II proteins the major conformational change within a protomer during membrane fusion is the relocation of domain III [20, 21, 33]. Our structural overlay analysis shows that PUUV sG_C_ is more structurally related to alphaviruses then to phleboviruses (S5A Fig). Furthermore, previous homology modeling studies used various alphavirus E1 proteins as a template for hantavirus G_C_ [39]. To generate a pre-fusion model for PUUV sG_C_ monomer, we therefore used SFV E1 protein as our reference model. Interestingly, the 1C9 epitope is exposed in our pre-fusion model while in the post-fusion structure it is protected by domain III (Fig 4). This suggests that binding of MAb 1C9 restricts domain III relocation and thus inhibits the fusion process. However, the multimerization arrangement of G_C_ on the virus envelope needs to be taken into account as this epitope might be partially or completely buried in the context of the mature virion. In contrast, the epitope of MAb 4G2 maps to domain II in proximity to the fusion loop. It was shown for PUUV that the neutralizing MAb 4G2 binds to G_C_ at neutral pH, however 4G2 does not recognized G_C_ that was exposed to low pH [39]. The 4G2 epitope was narrowed down to five residues that are sufficient for the antibody to bind and neutralize (Fig 4, dark yellow surface) [42]. Although this region of the epitope barely makes contacts with the neighboring protomer, the presence of a bound antibody will sterically hinder the formation of the trimer and thereby is expected to prevent fusion. Once a trimer is formed, the 4G2 antibody can no longer bind this epitope and therefore will not be reactive. Taken together, our structural epitope analysis and the disappearance of the 4G2 epitope below pH 6.2 [39] propose that the 4G2 MAb inhibit membrane fusion through interfering in trimer formation.

### Hinge-motions in the membrane proximal part of domain II

**Fig 5:**
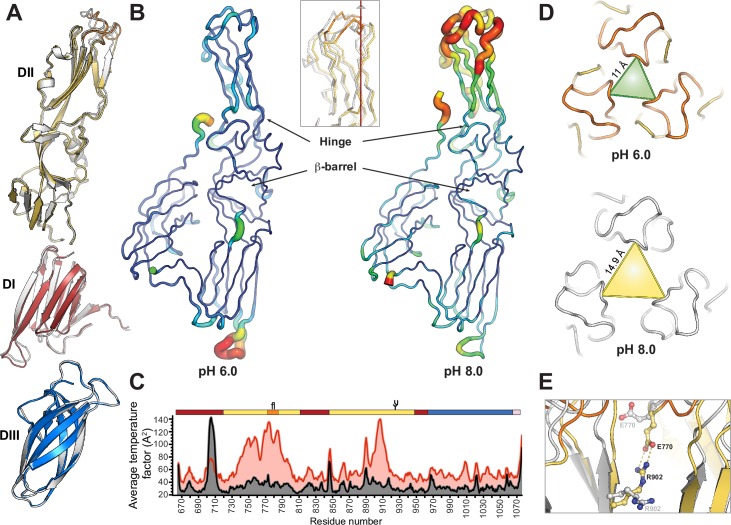
Hinge motions in PUUV sG_C_ protomers. (A) Superposition of individual domains of sG_C_^XF1^ (color) and sG_C_^XF2^ (light grey). Root mean square deviation (RMSD, calculated in PyMol) for domain I, II and III are 0.339 Å, 0.483 Å, 0.227 Å respectively. (B) B-factor putty representation of the two crystal structures of PUUV sG_C_. Cold colors (blue-green) represent lower B-factors whereas warm colors (yellow-red) represent high B-factors. In the inset is a ribbon representation of sG_C_^XF1^ (color) and sG_C_^XF2^ (light grey) in the same orientation as the putty representation. In red is the crystallographic 3-fold axis. (C) A quantifying B-factor analysis of the two PUUV sG_C_ crystal forms. Analysis was executed using bavarage module in CCP4 program suite (61). In black is sG_C_^XF1^ and red is sG_C_^XF2^. Linear domain organization is shown for orientation. Color scheme and domains borders are as in Fig 1. (D) A view on the fusion loop down the three-fold axis of sG_C_^XF1^ (color) and sG_C_^XF2^ (light grey) superposition. Triangles represent the distances between the C_α_^W773^ of the protomers. The distance in sG_C_^XF1^ (pH 6.0) is 11.0 Å whereas in sG_C_^XF2^ (pH 8.0) it is 14.9 Å. Triangles area for pH 6.0 and pH 8.0 are 52.4 Å^2^ and 114.1 Å^2^, respectively. (E) E770-R902 inter-protomer salt bridge at the two crystal forms. Color scheme is as in panel B.

It has been shown that in class II membrane fusion proteins there is a hinge motion between domain I and II [reviewed in [19]], and mutations at that region affect the pH threshold for fusion. However it seems that in phlebovirus G_C_ this region is more rigid [25]. As mentioned before, we also obtained crystals of PUUV sG_C_ at pH 8.0 (sG_C_^XF2^, see Table 1). Intriguingly, despite the slightly basic pH of the crystallization condition, sG_C_ still adopted the post-fusion conformation and assembles as trimers around the crystallographic 3-fold axis, however in a different space group lattice (Table 1). Although individual domains superposition did not reveal significant differences (Fig 5A) we still observed some noteworthy differences in the post-fusion structure of PUUV sG_C_^XF2^, particularly in the membrane proximal part of domain II including the fusion loop. In sG_C_^XF2^ this region has higher B-factor values than in the crystal form obtained at pH 6.0 (Fig 5B and 5C). Domain II undergoes a hinge motion of 4.5° away from the three-fold axis in the C_α_ backbone with respect to the sG_C_^XF1^ structure, increasing the distances between the fusion loops by approximately 35% (Fig 5D). Intriguingly, in sG_C_^XF2^, E770 and R902 adopt different rotamers that do not allow the salt bridge to form that is in contrast to the β-barrel at the domain I-II interface which limits the hinge motion at that region, unlike other class II membrane fusion proteins, but similar to RVFV G_C_ (Fig 5A) [25]. The absence of this inter-protomer salt-bridge plausibly contributes to the flexibility of the trimer at domain II membrane proximal region in the sG_C_^XF2^ structure (Fig 5E). However the unaffected fusion activity of the R902A in our functional assay suggests that it is not mandatory for fusion activity (S4B Fig). Indeed it was suggested before that there is no preferred distance between fusion loops of class II proteins required for fusion activity [49]. Finally, it was postulated that histidine residues function as pH sensors in class II membrane fusion proteins from flaviviruses [50–53]. We did not observe any significant differences in rotamers of histidine residues between the low and high pH crystal form. Furthermore, the poor sequence similarity between PUUV G_C_ (post fusion) and RVFV G_C_ (pre-fusion) shows no conserved histidines neither in sequence nor in three-dimension position (S5B Fig) implying that pH-sensing mechanism might be different in these two viruses.

### The stem region stabilizes the PUUV G_C_ post-fusion trimer

The total length of the stem region connecting between domain III and the TM domain is 46 residues (1069–1114) (S2 Fig). To maximize solubility we included in our expression construct just the first 38 residues of the stem. However, in both our crystal structures (sG_C_^XF1^ and sG_C_^XF2^) only the first eight residues of the stem (1069–1076) are visible in the electron density map, indicating either major flexibility or proteolytic cleavage at the C-terminus of sG_C_ during preparation. Unlike flaviviruses, in which the stem has an α-helical structure [54], or rubella virus in which the stem has a mixed α/β secondary structure content [34], secondary structure prediction of the stem region from PUUV G_C_ shows mostly random coil structure with a few residues predicted to be in β-strand conformation towards the TM domain (S2 Fig, pink/gray arrow). This might resemble the rubella E1 C-terminal β-strand ‘n’ as it joins the *i-j* β-sheet of a neighboring protomer [34]. It is possible that the C-terminal part of the stem region of PUUV G_C_ might extend the *i-j* β-sheet from domain II of the adjacent protomer and thereby might enhance the stability of the trimer.

**Fig 6:**
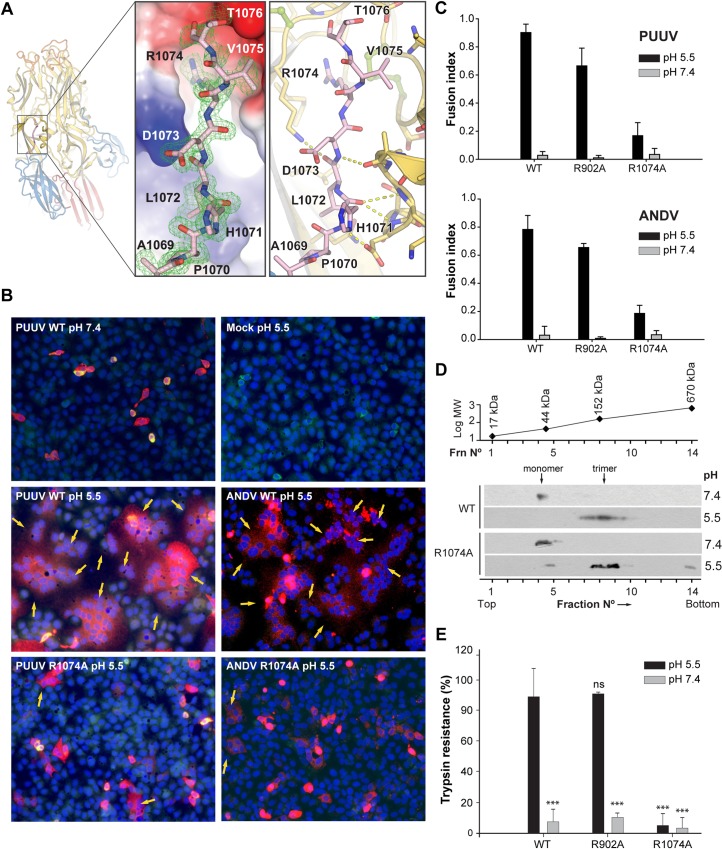
The stem region stabilizes the G_C_ trimer. (A) On the left an orientation overview of PUUV G_C_ trimer. In the middle, a close-up view of the stem region of one of the protomers. Side-chains of the stem residues are shown in sticks representation. The surface electrostatic potential (red, -5 kT/e; blue, 5 kT/e) of domain II was calculated by APBS [72]. 2F_O_-F_C_ electron density map at 1σ is shown in green mesh. On the right is a detailed view of the interactions of the stem region in sticks/cartoon representation. Color scheme is as in Fig 1. (B) Cell-cell fusion activity of wild type and mutant G_C_ from PUUV and ANDV G_C_. Representative fluorescence micrographs of Vero E6 cells expressing wild type or R1074A mutant GPC from PUUV or ANDV, and treated at different pHs. The cell cytoplasm was labelled with 5-chloromethylfluorescein diacetate (CMFDA; green fluorescence), nuclei with DAPI (blue fluorescence) and G_C_ was detected with anti- G_C_ MAb (Alexa555; red fluorescence). Cells from a partial microscopy field are shown from a representative experiment. Mock indicates cells transfected with an empty expression plasmid. Arrows indicate syncytia. (200 x magnification). (C) Quantification of the cell-cell fusion activity of cells expressing wild type and mutant GPC. The mean fusion index was calculated by counting cells and nuclei and represents n ≥ 2 independent experiments. Fusion activity of G_C_ mutant R902A was similar to the wild type and serves as a positive control. (D) Homotrimer formation of wild type and R1074A mutant Gc from ANDV after low pH treatment. Sucrose gradient sedimentation of glycoproteins extracted from ANDV-like particles after their treatment at the indicated pHs. Detection of G_C_ in each fraction by western blot using anti-G_C_ MAb. The molecular mass of each fraction was determined experimentally by a molecular marker and plotted against the log of its theoretical molecular mass. G_C_ trimers have a molecular mass of 165 KDa. (E) Trimer stability of wild type or mutant G_C_. VLPs including wild type G_N_ and wild type or mutant G_C_ were treated at different pHs and next incubated for 30 min with trypsin. The trypsine resistance of G_C_ was assessed by western blot analysis with anti-G_C_ MAb. Results were quantified by densitometry from n ≥ 2 experiments. As a control, the fusion active mutant R902A serves as trypsin-resistant control. The statistical evaluation of each data point was performed in relation to the wild type G_C_ treated at pH 5.5. ***, P < 0.00025; **, P < 0.0025;*, P < 0.025; ns, not significant.

Most of the inter- and intramolecular contacts at that region of the stem of PUUV sG_C_ are either main-chain/main-chain or main-chain/side-chain interactions (Fig 6A). Interestingly, R1074 side chain at the N-terminal of the stem is inserted into a negatively charged cavity at the same protomer (Fig 6A). The main-chain carbonyls of G883, D884, K893 and C894 create the cavity’s negative charge and lead the stem to a canyon formed by two adjacent protomers (Fig 6A). In flaviviruses the domain III-proximal part of the stem participates in both, intramolecular contacts with domain II and intermolecular interactions with the adjacent protomer, in what that appears to be a late-stage fusion intermediate [55]. The resemblance of our stem region orientation to flavivirus E stem implies the same for PUUV G_C_.

The stem region’s sequence is conserved among hantaviruses (S2 Fig). A recently published work exploring the stem region characteristics in ANDV showed inhibition of fusion activity for stem peptides derived from the C-terminal half of the stem region but not for peptides that were derived from the N-terminal half (domain III-proximal) [56]. The nature of the stem interactions with domain II observed in our structure might explain a weak binding of such exogenous peptides. Nevertheless, the zipper-like contact that we observed for residues 1069–1076 is evidently strong enough to immobilize a covalently attached stem segment but apparently not to bind a soluble peptide. In Semliki forest virus (genus: *Alphavirus*) it was shown that no specific sequence of the stem region was required for membrane fusion [57]. R1074 (R417 in Gc numbering) is the only residue at the base of PUUV sG_C_ stem that maintains side chain intramolecular contacts with domain II and it is highly conserved among hantaviruses (except HNTV and SEOV where it is substituted with lysine of similar properties, S2 Fig). To investigate the role of R1074 in membrane fusion, we introduced an alanine substitution of R1074 in both, PUUV and ANDV GPC in order to test their activity in the available *in vitro* systems established mostly for ANDV [17, 58]. The R1074A mutants of ANDV and PUUV G_C_ were expressed as the wild type proteins, localized on the cell surface and assembled into virus like particles (VLPs) (S4A Fig). However, despite being present on the cell surface, we found that the fusion index of the R1074 mutants from PUUV and ANDV dropped below 0.2, indicating a strong impairment of the acid pH-triggered syncytia formation activity (Fig 6B and 6C). The fact that the mutation of a conserved residue such as R1074 in hantavirus G_C_ from PUUV and ANDV led to equivalent fusion activity results provides a direct proof for its high conservation among hantaviruses in both, structure and function. Therefore, this data imply that the PUUV G_C_ structure can be used for rational design and characterization of mutations in different hantaviruses. In this context, and to further assess mechanistically the stage in which the R1074A mutant was arrested in the fusion process, we used the ANDV system to test acid-induced trimerization. Therefore, the wild type or R1074A mutant G_C_ from ANDV was incorporated together with wild type G_N_ into VLPs, that were collected and concentrated from the supernatants of cells expressing ANDV wild type or R1074A mutant GPC (S4 Fig). The concentrated VLPs were then treated at pH 7.4 or pH 5.5 and the glycoproteins subsequently extracted by non-ionic detergent. Their sedimentation on sucrose gradients revealed that the R1074A mutant underwent trimerization at pH 5.5 as efficient as the wild type control (Fig 6D). However, when the resistance of the trimer was tested for its stability by trypsin digestion, not only the neutral pH form, but also the acid-treated R1074A mutant was readily degraded by trypsin, in contrast to the low pH form of wild type G_C_ (Fig 6E). From these data it can be concluded that the R1074A mutant underwent acid-induced trimerization, but this trimer did not reach a stable post-fusion conformation. This difference in stability may be related to an incomplete fold-back of the stem region against the trimeric core. Combining our structural and functional data we conclude that the ‘base’ of the stem region in hantaviruses is essential for fusion through the formation of a stable post-fusion trimer.

It was shown previously for class II membrane fusion proteins that the activity of small molecule inhibitors in an assay for infectivity correlates well with their capacity to compete with stem-derived peptides [59]. Schmidt and co-workers suggested that the conformational transition from a pre-fusion arrangement to a post-fusion trimer will require removal of the ligand, imposing a barrier to completion of the fusion process. For this reason, *in silico* screens found potential pocket-binding compounds, that in some cases yielded active inhibitors [60–63]. Thus the electrostatic interaction of R1074 in a well-defined cavity at the base of the stem region and our functional data showing its role in trimer stabilization and membrane fusion activity suggest that this cavity might be a target for small molecule fusion inhibitors.

### Evolutionary implications of the PUUV sG_C_ structure

**Fig 7:**
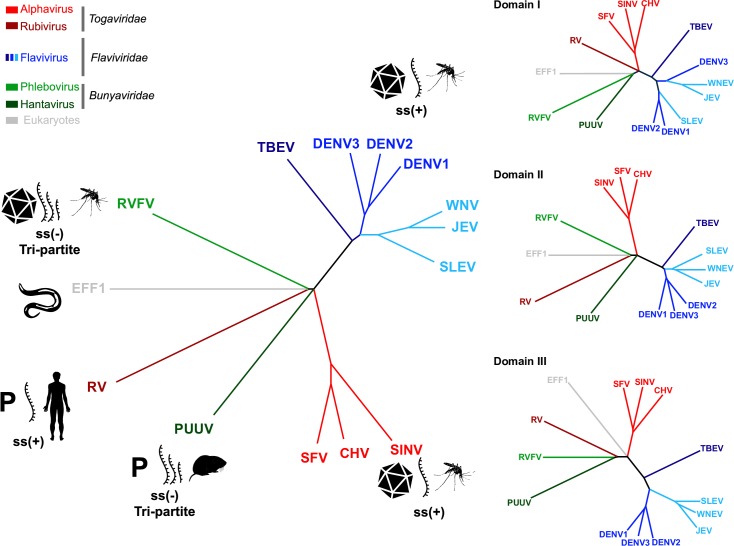
Structural and evolutionary relationships in class II fusion proteins. Cladograms representing the structural relationships between different class II fusion proteins. The coordinates of PUUV G_C_ were submitted to the DALI server. Atomic coordinates were obtained from the Protein Data Bank (PDB). Structure based alignment of the collected coordinates was performed with MUSTANG. A dendrogram was estimated based on a neighbor-joining analysis of the aligned sequences and guided by the BLOSUM62 substitution matrix. Abbreviations and their respective PDBs are as follows: dengue virus 2 (DENV-2, 1OK8-A), tick-borne encephalitis virus (TBEV, 1SVB-A), dengue virus 3 (DENV-3, 1UZG-A), Semliki forest virus (SFV, 1RER-A), West Nile virus (WNV, 2I69-A), Dengue virus 1 (DENV-1, 3G7T-A), Sindbis virus (SINV, 3MUU-A), Chikugunya virus (CHV, 3N41-F), Japanese encephalitis virus (JEV, 3P54-A), rubella virus (RV, 4ADG-A), St. Louis encephalitis virus (SLEV, 4FG0-A), Rift valley fever virus (RVFV, 4HJ1). *C*.*elegans* EFF1 (4OJC-A) was added as an out-group. Color scheme is per legend. Icosahedron icon represents icosahedral envelope, **P**- pleomorphic envelope. Genome type and transmission vectors are indicated by representative symbols.

The existence of a class II fold in a virus family other than *Flaviviridae* and *Togaviridae* was already suggested to diverge either from a viral or a common cellular class II ancestor [25, 35, 64, 65]. What are the driving forces that shaped the evolution of class II membrane fusion proteins? To address this question we computed structure-based sequence alignment based on both, the full-length ectodomains and the individual domains of various class II membrane fusion protein structures and calculated the corresponding cladograms (Fig 7). As expected, cladograms based on the structures of the individual domains do not show significant difference in topology compared to the full-length-based cladogram. Despite the structural similarities of phlebovirus G_C_ to flavivirus E proteins [25], PUUV G_C_ seems to be more structurally related to alphavirus E1 proteins (S5A Fig). On the other hand, rubella virus (RV) E1 and PUUV G_C_ appear to be more related in terms of the particle arrangement. Both assemble into pleomorphic virions with a non-continuous protein envelope with local symmetry properties in contrast to other viral class II membrane fusion proteins that are part of an icosahedral envelope arrangement [12, 34]. Furthermore, while the viruses containing class II membrane fusion proteins assembled into icosahedral symmetry are all arthropod-borne, hantaviruses and RV are transmitted among mammals (rodent-to-human and human-to-human, respectively). As suggested before for RV, a human-restricted infection cycle forced the virus to evolve unique structural features for its fusogen [34]. It is possible that hantaviruses followed a similar evolutionary path in mammals and further diverged to an additional branch separated from arboviruses containing class II membrane fusion proteins (Fig 7). Nonetheless, other evolutionary mechanisms such as convergent evolution, cannot be ruled out for this observation. Hopefully with the determination of more fusogens structures from the *Bunyaviridae* family the molecular basis for these proteins evolution will be more comprehensively studied.

## Materials and Methods

### Protein expression and purification

The open reading frame encoding the ectodomain of G_C_ (sG_C_) from PUUV (M segment residues 659–1106) were amplified from the M segment cDNA of Puumala virus P360 strain (GenBank accession code P41266.1) and subcloned into the pAcGP67 vector (BD Biosciences) in frame with the baculovirus gp67 signal sequence and a C-terminal eight-histidine purification tag. Sf9 insect cells (Expression Systems) were co-transfected with sG_C_ expression constructs and linearized baculovirus genomic DNA (Expression Systems) to produce recombinant baculoviruses expressing sG_C_. Virus stocks were amplified with three sequential infections of Sf9 cells. For sG_C_ expression, *Tni* insect cells (Expression Systems) grown at 27°C were infected at a density of 2 × 10^6^ cells/ml with 1% (v/v) of third-passage (P3) baculovirus stock. After culture in suspension for 96–108 h at 20°C the culture media was collected and its pH was adjusted with addition of Tris pH 8.0 to final concentration of 20 mM. Following media concentration, secreted sG_C_ was purified by nickel affinity chromatography (Ni-NTA agarose, QIAgen). A subsequent anion-exchange chromatography purification step (monoQ, GE Healthcare) resulted in two populations of sG_C_ eluting in different salt concentrations. From this point on the two populations (termed sG_C_^XF1^ and sG_C_^XF2^) were separated and further went through the same steps. The His-tag was subsequently removed with carboxypeptidase A (CPA) treatment at 4°C for 16 h (1 mU CPA per microgram of sG_C_). CPA was then inhibited with 1 mM EDTA and 1 mM 1,10-phenanthroline and separated from sG_C_ by size-exclusion chromatography (Superdex 200 10/300 GL, GE Healthcare). Protein samples were concentrated to 2.5–3.5 g/l, frozen in liquid nitrogen and stored at -80°C in 10 mM Tris pH 8, 0.1 M NaCl.

### Crystallization and structure determination of sG_C_

Crystals of sG_C_^XF1^ (eluted from the mono-Q at low salt concentration) were grown by hanging drop vapor diffusion at 16°C. sG_C_^XF1^ at 2.4 g/l in 10 mM Tris pH 8.0, 0.1 M NaCl was mixed in 2:1 protein to reservoir containing 12% (w/v) polyethylene glycol 2000 mono-methyl ether (PEG 2000 MME), 0.1 M MES pH 6.0 and 0.2 M ammonium sulfate. Multi-crystals clusters appeared after 3–5 weeks and very few single crystals were observed after 6–8 weeks. A single crystal was then crushed and used as microseeds in drops pre-equilibrated for 24 h prior to seeding. Rhombohedron shaped crystals reached a size of 150 × 70 × 70 μm 7–10 days post-seeding and belonged to space group *R32*. Crystals were frozen in liquid nitrogen in reservoir solution supplemented with 30% PEG 400 as a cryoprotectant. Derivative sG_C_^XF1^ crystals were obtained by soaking in reservoir solution plus 1 mM methyl mercury phosphate (Hampton Research) for one week. sG_C_^XF2^ crystals appeared in 40% (w/v) polyethylene glycol 400 (PEG 400), 0.1 M Tris pH 8.0, 0.2 M lithium sulfate (1:1 protein to reservoir ratio). After 12 weeks sharp-edges cubic crystals were observed and reached a size of 60 × 60 × 60 μm. Upon optimization, crystals with cubic morphology at the size of 75 × 75 × 75 μm appeared after 4–6 weeks and belonged to space group I2_1_3. Data were collected at 100 K on a PILATUS detector (Dectris) and processed with XDS [66]. The structure of sG_C_^XF1^ was determined by single isomorphous replacement with anomalous signal (SIRAS) with PHENIX [67]. Initial atomic coordinates for sG_C_^XF1^ built with PHENIX were used as starting model in refinement and building cycles with the highest resolution (1.84 Å) native data set. The atomic model was completed with COOT [68] and refined to an *R*_*free*_ of 21% with PHENIX and REFMAC [69]. The structure of sG_C_^XF2^ was determined by molecular replacement using domains I+III and domain II of sG_C_^XF1^ as separate search models. Atomic coordinates and structure factors for sG_C_^XF1^ and sG_C_^XF2^ have been deposited in the Protein Data Bank (ID codes 5J81and 5J9H, respectively). See Table 1 for data collection and refinement statistics. All molecular graphics were produced using PyMol (PyMOL Molecular Graphics System, Version 1.8 Schrödinger, LLC). Molecular surface calculations were performed using UCSF Chimera [70]. Surface electrostatic potential was calculated with APBS [71]. B-factor analysis was calculated using baverage module in the CCP4 suite [72]. Surface conservation was calculated using CONSURF server (http://consurf.tau.ac.il/) [73].

### PUUV sG_C_ pre-fusion conformation modeling

PUUV sG_C_ structure was superimposed on the crystal structure of Semliki forest virus E1 in its pre-fusion state (PDB ID codes 2ALA) using domains I+II and domain III as two separate rigid bodies. The flexible linker between domain I and domain III was eliminated from the model.

### Hydrodynamic and multiangle scattering analysis

Analytical size-exclusion chromatography and multiangle light scattering (MALS) experiments were performed in 20 mM sodium acetate pH 5.0, or Tris⋅HCl pH 8.0 and 0.1 M NaCl. A total of 0.2 mL sG_C_ at 2.5 g/L was loaded onto a Superdex 200 (10/300) column coupled to mini DAWN TREOS spectrometer and Optilab T-rEX (Wyatt technology) refractometer at a flow rate of 0.7 mL/min. PUUV sG_C_ was detected as it eluted from the column with a UV detector at 280 nm, a light scattering detector at 690 nm, and a refractive index detector. The molar mass of PUUV sG_C_ was determined from the Debye plot of light scattering intensity versus scattering angle. Data processing was performed with ASTRA software (Wyatt Technology).

### Expression, cell surface localization of mutant Gc and assembly into VLPs

Mutations were introduced into the expression vectors pI.18/ANDV-GPC [74] and pWRG/PUUV-M(s2) (kindly provided by Jay Hooper, USAMRIID, USA) [75] coding for GPC from ANDV strain Chi-7913 and PUUV strain K27 (GenBank accession numbers AAO86638 and L08754), respectively, by using DNA synthesis and sub-cloning into the corresponding expression vectors (Genscript). For expression and localization analysis, 8 μg of plasmids were calcium-transfected into 293FT cells (Invitrogen) grown on 100 mm plates and 48 hrs later, proteins located on the cell surface were biotinylated using a cell-surface protein isolation kit (Pierce), and the fractions corresponding to intracellular and surface proteins separated on a neutravidin resin. The presence of Gc and β-actin in each fraction were analyzed by western blot using anti-Gc 2H4/F6 [76] and anti-β-actin (Sigma) MAb at a 1:2,500 dilution. Primary antibodies were detected by chemiluminsecence using anti-mouse immunoglobulin horseradish peroxidase conjugate (Thermo Fisher Scientific). To prepare VLPs, a previously established protocol was used [17]. Briefly, 48 hrs post-transfection the supernatant of 293FT cells transfected with wild type or mutant pI.18/ANDV-GPC or pWRG/PUUV-M(s2) constructs was collected and VLPs concentrated by ultracentrifugation for 1 hr at 135,000 g. The presence of VLPs was assayed by western blot analysis as described above.

### Cell-cell fusion activity of mutant Gc

A fluorescence-based syncytia assay was performed as reported before (32). Vero E6 cells (ATTC) seeded in 16-well chamber slides were transfected with 0.5 μg of wild type or mutant pI.18/ANDV-GPC or pWRG/PUUV-M(s2) constructs using lipofectamin 2000 (Invitrogen). 48 hrs later, the cells were incubated for 5 min at 37°C with MEM culture media adjusted to the corresponding pH. Next, incubation of cells was continued for 3 hrs at 37°C in neutral pH MEM culture media. To label the cell cytoplasm, cells were subsequently incubated for one hr with 1 μM 5-chloromethylfluorescein diacetate (Cell Tracker CMFDA, Molecular Probes). Subsequently, cells were fixed with 4% (w/v) paraformaldehyde, permeabilized with 0.1% (v/v) Triton X-100 and Gc detected with anti-Gc 2H4/F6 MAb and anti-mouse immunoglobulin MAb Alexa555 conjugate (Invitrogen). Cell nuclei were stained with DAPI 1 ng/μl in PBS. To visualize syncytia samples were examined under a fluorescence microscope (BMAX51; Olympus) and pictures taken for quantification (ProgRes C3; Jenoptics). The fusion index of Gc-expressing cells was calculated using the formula: 1- [number of cells/number of nuclei]. For each sample approximately 200 nuclei per field were counted (200 x magnification) and the mean fusion index of five fields calculated from at least two independent experiments.

### Multimerization analysis of mutant Gc

Acid-induced Gc trimerization was tested by sucrose sedimentation using a previous protocol [17]. Briefly, VLPs were incubated for 30 min at the indicated pH to allow for Gc conformational changes. The pH back-neutralized, and Triton X-100 (0,5%; v/v)-extracted glycoproteins were subsequently loaded to the top of a sucrose step gradient (7–15%, w/v). After 16 hrs of centrifugation at 150,000 g, fractions were collected and the presence of Gc in each fractions tested by western blot analysis.

### Trypsin resistance of mutant Gc

The stability of neutral pH and acid pH conformation of a Gc mutant was assayed by its resistance to trypsin as shown for wild type Gc previously [17]. In brief, VLPs including wild type or mutant Gc were incubated at the indicated pH and presence of Gc assessed by western blot as described above.

### Class II membrane fusion protein cladogram construction

A set of structures of class II fusion proteins in their post fusion conformation was obtained from the DALI server [77] with the atomic coordinates of PUUV sG_C_ as the query. Structures of viral class II fusion proteins and of *C*.*elegans* EFF-1 were aligned with the MUSTANG server [78]. The resulting structure-based sequence alignment was used for the estimation of the cladogram by the neighbor-joining method with the BLOSUM62 substitution matrix using Jalview [79, 80]. The same process was further executed on individual domains.

## Supporting Information

S1 Fig Comparison of PUUV sG_C_ with other class II proteins in their post-fusion conformation. From left: crystal structures of PUUV sG_C_, Semliki forest virus E1 (PDB entry 1RER), Rubella virus E1 (PDB entry 4ADI) and Dengue virus glycoprotein E (PDB entry 1OK8) in their post-fusion conformation. To simplify, only one protomer from each trimer is shown.
(TIF)

S2 Fig Multiple sequence alignment (MSA) of human pathogenic hantaviruses. Amino acid sequence alignment of G_C_ from selected hantaviruses. Conserved residues were replaced with periods. Domain colors are as in Fig 1. Arrows denote β-strands and cylinders represent α-helices. Glycans are represented by cyan hexagons and disulfide bonds are indicated in green. The unoccupied glycosylation site is represented with single grey hexagon. Light pink and gray shading regions corresponds the unmodeled stem and transmembrane regions, respectively. Cytoplasmic C-terminal tail presented with no shading. Secondary structure prediction of the C-terminal β-strand is represented by a pink/grey arrow. Numbered green circles represent cysteine residues, where cysteine residues with the same numbering are disulfide linked. Black bars indicate neutralizing epitopes. Database sequence accession codes are per legend and correspond to the Uniprot database (http://www.uniprot.org).
(TIF)

S3 Fig PUUV sG_C_ is a monomer in solution. (A) A total of 0.2 mL of sG_C_ (1 g/L) was loaded onto a Superdex 200 (30/100) size-exclusion column pre-equilibrated with 20 mM Tris buffer pH 8.0 and 100 mM NaCl. The eluate was analyzed for absorbance at 280 nm. Inset: Standard curve obtained with proteins of known masses. The position of G_C_ on the curve is indicated with an arrow. The corresponding MW of sG_C_ was calculated using the line equations of a standard curve. The MW of sG_C_ calculated from the sequence is 49.3 KDa excluding glycosylations. On the right, a Coomassie stained SDS-PAGE analysis of the two preparations. (B) SEC-MALS analysis of PUUV G_C_ in different pHs. 0.2 mL at 2.5 g/L were loaded onto Superdex 200 column at pH 8.0 and pH 5.0. The elution was analyzed for absorbance at 280 nm (*right y* axis) and for multiangle light scattering, which was converted to molecular mass (*Left y* axis; material and methods). Gray rectangle represents the Mw range between 50–60 KDa. Colors are as per legend.
(TIF)

S4 Fig Cellular localization and cell-cell fusion activity of wild type and mutant G_C_ from PUUV and ANDV G_C_. (A) Western blot analysis of the presence of G_C_ in different cellular fractions and the supernatant of 293 FT cells expressing wild type or mutant GPC from PUUV and ANDV. Fractions correspond to non-biotinylated intracellular fraction, biotinylated cell surface fraction and the concentrated supernatant of cells. (B) Representative fluorescence micrographs of Vero E6 cells expressing wild type or R902A mutant GPC from PUUV or ANDV, and treated at different pHs. The cell cytoplasm was labelled with 5-chloromethylfluorescein diacetate (CMFDA; green fluorescence), nuclei with DAPI (blue fluorescence) and G_C_ was detected with anti-G_C_ MAb (Alexa555; red fluorescence). Cells from a partial microscopy field are shown from a representative experiment. Mock indicates cells transfected with an empty expression plasmid. Arrows indicate syncytia. (200 X magnification). Quantitative analysis of these cell-cell fusion assays is presented also in Fig 6C.
(TIF)

S5 Fig **Structural alignment and comparison of PUUV with other class II membrane fusion proteins** (A) PUUV G_C_ shows more structural similarity to alphaviruses then to other class II proteins. Table represents the DALI server (http://ekhidna.biocenter.helsinki.fi/dali_server/start) scores with PUUV G_C_ as the query. Z-score describe the statistical significance of a pairwise comparison score (higher score represents higher similarity), n/nt is the ratio between the number of aligned residues (n) and total residues in the structure (nt), σ is the Root mean square deviation (RMSD) for the aligned residues and % represents sequence identity. *Bunyaviridae* are in greens, *Togaviridae* in reds, *Flaviviridae* in blues and eukaryotes are in grey. (B) Sequence alignment of PUUV and RVFV G_C_ proteins. Alignment was obtained using MAFFT [81] the secondary structure assignment for RVFV was based on PDB entry 4HJ1. Colors scheme is as in Fig 1.
(TIF)

## References

[1] Hallin GW, Simpson SQ, Crowell RE, James DS, Koster FT, Mertz GJ, et al Cardiopulmonary manifestations of hantavirus pulmonary syndrome. Critical care medicine. 1996;24(2):252–8. 8605797 10.1097/00003246-199602000-00012

[2] Hughes MT, Gonzalez JA, Reagan KL, Blair CD, Beaty BJ. Comparative potential of Aedes triseriatus, Aedes albopictus, and Aedes aegypti (Diptera: Culicidae) to transovarially transmit La Crosse virus. J Med Entomol. 2006;43(4):757–61. Epub 2006/08/09. 16892636 10.1603/0022-2585(2006)43[757:cpoata]2.0.co;2

[3] Lee HW, Lee PW, Johnson KM. Isolation of the etiologic agent of Korean Hemorrhagic fever. The Journal of infectious diseases. 1978;137(3):298–308. 24670 10.1093/infdis/137.3.298

[4] Svedmyr A, Lee HW, Berglund A, Hoorn B, Nystrom K, Gajdusek DC. Epidemic nephropathy in Scandinavia is related to Korean haemorrhagic fever. Lancet. 1979;1(8107):100. 10.1016/s0140-6736(79)90083-7 84099

[5] Brummer-Korvenkontio M, Vaheri A, Hovi T, von Bonsdorff CH, Vuorimies J, Manni T, et al Nephropathia epidemica: detection of antigen in bank voles and serologic diagnosis of human infection. The Journal of infectious diseases. 1980;141(2):131–4. 6102587 10.1093/infdis/141.2.131

[6] Ettinger J, Hofmann J, Enders M, Tewald F, Oehme RM, Rosenfeld UM, et al Multiple synchronous outbreaks of Puumala virus, Germany, 2010. Emerging infectious diseases. 2012;18(9):1461–4. PubMed Central PMCID: PMCPMC3437711. 10.3201/eid1809.111447 22932394 PMC3437711

[7] Ali HS, Drewes S, Weber de Melo V, Schlegel M, Freise J, Groschup MH, et al Complete genome of a Puumala virus strain from Central Europe. Virus Genes. 2015;50(2):292–8. 10.1007/s11262-014-1157-6 25543297

[8] Ali HS, Drewes S, Sadowska ET, Mikowska M, Groschup MH, Heckel G, et al First molecular evidence for Puumala hantavirus in Poland. Viruses. 2014;6(1):340–53. PubMed Central PMCID: PMCPMC3917447. 10.3390/v6010340 24452006 PMC3917447

[9] Spiropoulou CF, Goldsmith CS, Shoemaker TR, Peters CJ, Compans RW. Sin Nombre virus glycoprotein trafficking. Virology. 2003;308(1):48–63. 12706089 10.1016/s0042-6822(02)00092-2

[10] Shi X, Elliott RM. Golgi localization of Hantaan virus glycoproteins requires coexpression of G1 and G2. Virology. 2002;300(1):31–8. 12202203 10.1006/viro.2002.1414

[11] Lober C, Anheier B, Lindow S, Klenk HD, Feldmann H. The Hantaan virus glycoprotein precursor is cleaved at the conserved pentapeptide WAASA. Virology. 2001;289(2):224–9. 10.1006/viro.2001.1171 11689045

[12] Huiskonen JT, Hepojoki J, Laurinmaki P, Vaheri A, Lankinen H, Butcher SJ, et al Electron cryotomography of Tula hantavirus suggests a unique assembly paradigm for enveloped viruses. Journal of virology. 2010;84(10):4889–97. Epub 2010/03/12. 10.1128/JVI.00057-10 20219926 PMC2863824

[13] Li S, Rissanen I, Zeltina A, Hepojoki J, Raghwani J, Harlos K, et al A Molecular-Level Account of the Antigenic Hantaviral Surface. Cell Rep. 2016. 10.1016/j.celrep.2016.06.039 PMC5638784 27355863

[14] Ogino M, Yoshimatsu K, Ebihara H, Araki K, Lee BH, Okumura M, et al Cell fusion activities of Hantaan virus envelope glycoproteins. Journal of virology. 2004;78(19):10776–82. PubMed Central PMCID: PMC516380. 10.1128/JVI.78.19.10776-10782.2004 15367644 PMC516380

[15] Jin M, Park J, Lee S, Park B, Shin J, Song KJ, et al Hantaan virus enters cells by clathrin-dependent receptor-mediated endocytosis. Virology. 2002;294(1):60–9. 10.1006/viro.2001.1303 11886265

[16] Ramanathan HN, Jonsson CB. New and Old World hantaviruses differentially utilize host cytoskeletal components during their life cycles. Virology. 2008;374(1):138–50. 10.1016/j.virol.2007.12.030 18234268

[17] Acuna R, Bignon E, Mancini R, Lozach PY, Tischler ND. Acidification triggers Andes hantavirus membrane fusion and rearrangement of Gc into a stable post-fusion homotrimer. The Journal of general virology. 2015. 10.1099/jgv.0.000269 26310672

[18] Lozach PY, Mancini R, Bitto D, Meier R, Oestereich L, Overby AK, et al Entry of bunyaviruses into mammalian cells. Cell Host Microbe. 2010;7(6):488–99. Epub 2010/06/15. 10.1016/j.chom.2010.05.007 20542252 PMC7172475

[19] Modis Y. Class II fusion proteins. Adv Exp Med Biol. 2013;790:150–66. 10.1007/978-1-4614-7651-1_8 23884590 PMC7123093

[20] Gibbons DL, Vaney MC, Roussel A, Vigouroux A, Reilly B, Lepault J, et al Conformational change and protein-protein interactions of the fusion protein of Semliki Forest virus. Nature. 2004;427(6972):320–5. Epub 2004/01/23. 10.1038/nature02239 14737160

[21] Modis Y, Ogata S, Clements D, Harrison SC. Structure of the dengue virus envelope protein after membrane fusion. Nature. 2004;427(6972):313–9. Epub 2004/01/23. 10.1038/nature02165 14737159

[22] Skehel JJ, Wiley DC. Receptor binding and membrane fusion in virus entry: the influenza hemagglutinin. Annu Rev Biochem. 2000;69:531–69. Epub 2000/08/31. 10.1146/annurev.biochem.69.1.531 10966468

[23] Tischler ND, Gonzalez A, Perez-Acle T, Rosemblatt M, Valenzuela PD. Hantavirus Gc glycoprotein: evidence for a class II fusion protein. The Journal of general virology. 2005;86(Pt 11):2937–47. 10.1099/vir.0.81083-0 16227214

[24] Garry CE, Garry RF. Proteomics computational analyses suggest that the carboxyl terminal glycoproteins of Bunyaviruses are class II viral fusion protein (beta-penetrenes). Theor Biol Med Model. 2004;1:10 Epub 2004/11/17. 10.1186/1742-4682-1-10 15544707 PMC535339

[25] Dessau M, Modis Y. Crystal structure of glycoprotein C from Rift Valley fever virus. Proceedings of the National Academy of Sciences of the United States of America. 2013;110(5):1696–701. PubMed Central PMCID: PMCPMC3562824. 10.1073/pnas.1217780110 23319635 PMC3562824

[26] Kong L, Giang E, Nieusma T, Kadam RU, Cogburn KE, Hua Y, et al Hepatitis C virus E2 envelope glycoprotein core structure. Science. 2013;342(6162):1090–4. PubMed Central PMCID: PMCPMC3954638. 10.1126/science.1243876 24288331 PMC3954638

[27] Li Y, Wang J, Kanai R, Modis Y. Crystal structure of glycoprotein E2 from bovine viral diarrhea virus. Proceedings of the National Academy of Sciences of the United States of America. 2013;110(17):6805–10. PubMed Central PMCID: PMCPMC3637714. 10.1073/pnas.1300524110 23569276 PMC3637714

[28] Stiasny K, Bressanelli S, Lepault J, Rey FA, Heinz FX. Characterization of a membrane-associated trimeric low-pH-induced Form of the class II viral fusion protein E from tick-borne encephalitis virus and its crystallization. Journal of virology. 2004;78(6):3178–83. Epub 2004/03/03. 10.1128/JVI.78.6.3178-3183.2004 14990739 PMC353737

[29] Zhang X, Sheng J, Austin SK, Hoornweg TE, Smit JM, Kuhn RJ, et al Structure of acidic pH dengue virus showing the fusogenic glycoprotein trimers. Journal of virology. 2015;89(1):743–50. PubMed Central PMCID: PMCPMC4301137. 10.1128/JVI.02411-14 25355881 PMC4301137

[30] de Boer SM, Kortekaas J, Spel L, Rottier PJ, Moormann RJ, Bosch BJ. Acid-activated structural reorganization of the Rift Valley fever virus Gc fusion protein. Journal of virology. 2012. Epub 2012/10/05. 10.1128/JVI.01973-12 PMC3503025 23035232

[31] Zheng F, Ma L, Shao L, Wang G, Chen F, Zhang Y, et al Envelope glycoproteins of hantavirus can mediate cell-cell fusion independently. The new microbiologica. 2007;30(2):101–7. 17619252

[32] Cifuentes-Munoz N, Barriga GP, Valenzuela PD, Tischler ND. Aromatic and polar residues spanning the candidate fusion peptide of the Andes virus Gc protein are essential for membrane fusion and infection. The Journal of general virology. 2011;92(Pt 3):552–63. 10.1099/vir.0.027235-0 21123541

[33] Nayak V, Dessau M, Kucera K, Anthony K, Ledizet M, Modis Y. Crystal structure of dengue virus type 1 envelope protein in the postfusion conformation and its implications for membrane fusion. Journal of virology. 2009;83(9):4338–44. PubMed Central PMCID: PMC2668458. 10.1128/JVI.02574-08 19244332 PMC2668458

[34] DuBois RM, Vaney MC, Tortorici MA, Kurdi RA, Barba-Spaeth G, Krey T, et al Functional and evolutionary insight from the crystal structure of rubella virus protein E1. Nature. 2013;493(7433):552–6. 10.1038/nature11741 23292515

[35] Lescar J, Roussel A, Wien MW, Navaza J, Fuller SD, Wengler G, et al The Fusion glycoprotein shell of Semliki Forest virus: an icosahedral assembly primed for fusogenic activation at endosomal pH. Cell. 2001;105(1):137–48. 11301009 10.1016/s0092-8674(01)00303-8

[36] Rey FA, Heinz FX, Mandl C, Kunz C, Harrison SC. The envelope glycoprotein from tick-borne encephalitis virus at 2 A resolution. Nature. 1995;375(6529):291–8. Epub 1995/05/25. 10.1038/375291a0 7753193

[37] Modis Y, Ogata S, Clements D, Harrison SC. A ligand-binding pocket in the dengue virus envelope glycoprotein. Proceedings of the National Academy of Sciences of the United States of America. 2003;100(12):6986–91. Epub 2003/05/22. 10.1073/pnas.0832193100 12759475 PMC165817

[38] Kopaciewicz W, Regnier FE. Nonideal size-exclusion chromatography of proteins: effects of pH at low ionic strength. Analytical biochemistry. 1982;126(1):8–16. 7181119 10.1016/0003-2697(82)90102-6

[39] Hepojoki J, Strandin T, Vaheri A, Lankinen H. Interactions and oligomerization of hantavirus glycoproteins. Journal of virology. 2010;84(1):227–42. PubMed Central PMCID: PMCPMC2798430. 10.1128/JVI.00481-09 19828613 PMC2798430

[40] Wallin M, Ekstrom M, Garoff H. Isomerization of the intersubunit disulphide-bond in Env controls retrovirus fusion. The EMBO journal. 2004;23(1):54–65. PubMed Central PMCID: PMCPMC1271652. 10.1038/sj.emboj.7600012 14685283 PMC1271652

[41] Zheng F, Ma L, Shao L, Wang G, Chen F, Zhang Y, et al Defining the N-linked glycosylation site of Hantaan virus envelope glycoproteins essential for cell fusion. Journal of microbiology. 2007;45(1):41–7. 17342054

[42] Koch J, Liang M, Queitsch I, Kraus AA, Bautz EK. Human recombinant neutralizing antibodies against hantaan virus G2 protein. Virology. 2003;308(1):64–73. 12706090 10.1016/s0042-6822(02)00094-6

[43] Wang M, Pennock DG, Spik KW, Schmaljohn CS. Epitope mapping studies with neutralizing and non-neutralizing monoclonal antibodies to the G1 and G2 envelope glycoproteins of Hantaan virus. Virology. 1993;197(2):757–66. 10.1006/viro.1993.1652 7504368

[44] Liang M, Guttieri M, Lundkvist A, Schmaljohn C. Baculovirus expression of a human G2-specific, neutralizing IgG monoclonal antibody to Puumala virus. Virology. 1997;235(2):252–60. 10.1006/viro.1997.8695 9281505

[45] de Carvalho Nicacio C, Lundkvist A, Sjolander KB, Plyusnin A, Salonen EM, Bjorling E. A neutralizing recombinant human antibody Fab fragment against Puumala hantavirus. J Med Virol. 2000;60(4):446–54. 10686029 10.1002/(sici)1096-9071(200004)60:4<446::aid-jmv13>3.0.co;2-v

[46] Salonen EM, Parren PW, Graus YF, Lundkvist A, Fisicaro P, Vapalahti O, et al Human recombinant Puumala virus antibodies: cross-reaction with other hantaviruses and use in diagnostics. The Journal of general virology. 1998;79 (Pt 4):659–65. Epub 1998/05/06. 10.1099/0022-1317-79-4-659 9568958

[47] Lundkvist A, Horling J, Athlin L, Rosen A, Niklasson B. Neutralizing human monoclonal antibodies against Puumala virus, causative agent of nephropathia epidemica: a novel method using antigen-coated magnetic beads for specific B cell isolation. The Journal of general virology. 1993;74 (Pt 7):1303–10. 10.1099/0022-1317-74-7-1303 7687648

[48] Lundkvist A, Niklasson B. Bank vole monoclonal antibodies against Puumala virus envelope glycoproteins: identification of epitopes involved in neutralization. Archives of virology. 1992;126(1–4):93–105. 1381914 10.1007/BF01309687

[49] Luca VC, Nelson CA, Fremont DH. Structure of the St. Louis encephalitis virus postfusion envelope trimer. Journal of virology. 2013;87(2):818–28. PubMed Central PMCID: PMCPMC3554068. 10.1128/JVI.01950-12 23115296 PMC3554068

[50] Bressanelli S, Stiasny K, Allison SL, Stura EA, Duquerroy S, Lescar J, et al Structure of a flavivirus envelope glycoprotein in its low-pH-induced membrane fusion conformation. The EMBO journal. 2004;23(4):728–38. PubMed Central PMCID: PMC380989. 10.1038/sj.emboj.7600064 14963486 PMC380989

[51] Kanai R, Kar K, Anthony K, Gould LH, Ledizet M, Fikrig E, et al Crystal structure of west nile virus envelope glycoprotein reveals viral surface epitopes. Journal of virology. 2006;80(22):11000–8. PubMed Central PMCID: PMCPMC1642136. 10.1128/JVI.01735-06 16943291 PMC1642136

[52] Roussel A, Lescar J, Vaney MC, Wengler G, Wengler G, Rey FA. Structure and interactions at the viral surface of the envelope protein E1 of Semliki Forest virus. Structure. 2006;14(1):75–86. 10.1016/j.str.2005.09.014 16407067

[53] Mueller DS, Kampmann T, Yennamalli R, Young PR, Kobe B, Mark AE. Histidine protonation and the activation of viral fusion proteins. Biochem Soc Trans. 2008;36(Pt 1):43–5. 10.1042/BST0360043 18208382

[54] Zhang W, Chipman PR, Corver J, Johnson PR, Zhang Y, Mukhopadhyay S, et al Visualization of membrane protein domains by cryo-electron microscopy of dengue virus. Nat Struct Biol. 2003;10(11):907–12. PubMed Central PMCID: PMCPMC4148076. 10.1038/nsb990 14528291 PMC4148076

[55] Klein DE, Choi JL, Harrison SC. Structure of a dengue virus envelope protein late-stage fusion intermediate. Journal of virology. 2013;87(4):2287–93. PubMed Central PMCID: PMCPMC3571469. 10.1128/JVI.02957-12 23236058 PMC3571469

[56] Barriga GP, Villalon-Letelier F, Marquez CL, Bignon EA, Acuna R, Ross BH, et al Inhibition of the Hantavirus Fusion Process by Predicted Domain III and Stem Peptides from Glycoprotein Gc. PLoS Negl Trop Dis. 2016;10(7):e0004799 PubMed Central PMCID: PMCPMC4945073. 10.1371/journal.pntd.0004799 27414047 PMC4945073

[57] Liao M, Kielian M. Functions of the stem region of the Semliki Forest virus fusion protein during virus fusion and assembly. Journal of virology. 2006;80(22):11362–9. PubMed Central PMCID: PMCPMC1642169. 10.1128/JVI.01679-06 16971447 PMC1642169

[58] Acuna R, Cifuentes-Munoz N, Marquez CL, Bulling M, Klingstrom J, Mancini R, et al Hantavirus Gn and Gc glycoproteins self-assemble into virus-like particles. Journal of virology. 2014;88(4):2344–8. PubMed Central PMCID: PMC3911568. 10.1128/JVI.03118-13 24335294 PMC3911568

[59] Schmidt AG, Lee K, Yang PL, Harrison SC. Small-molecule inhibitors of dengue-virus entry. PLoS pathogens. 2012;8(4):e1002627 PubMed Central PMCID: PMCPMC3320583. 10.1371/journal.ppat.1002627 22496653 PMC3320583

[60] Yennamalli R, Subbarao N, Kampmann T, McGeary RP, Young PR, Kobe B. Identification of novel target sites and an inhibitor of the dengue virus E protein. J Comput Aided Mol Des. 2009;23(6):333–41. 10.1007/s10822-009-9263-6 19241120

[61] Wang QY, Patel SJ, Vangrevelinghe E, Xu HY, Rao R, Jaber D, et al A small-molecule dengue virus entry inhibitor. Antimicrob Agents Chemother. 2009;53(5):1823–31. PubMed Central PMCID: PMCPMC2681551. 10.1128/AAC.01148-08 19223625 PMC2681551

[62] Li Z, Khaliq M, Zhou Z, Post CB, Kuhn RJ, Cushman M. Design, synthesis, and biological evaluation of antiviral agents targeting flavivirus envelope proteins. J Med Chem. 2008;51(15):4660–71. PubMed Central PMCID: PMCPMC2562352. 10.1021/jm800412d 18610998 PMC2562352

[63] Kampmann T, Yennamalli R, Campbell P, Stoermer MJ, Fairlie DP, Kobe B, et al In silico screening of small molecule libraries using the dengue virus envelope E protein has identified compounds with antiviral activity against multiple flaviviruses. Antiviral Res. 2009;84(3):234–41. 10.1016/j.antiviral.2009.09.007 19781577

[64] Modis Y. Relating structure to evolution in class II viral membrane fusion proteins. Current opinion in virology. 2014;5:34–41. PubMed Central PMCID: PMC4028412. 10.1016/j.coviro.2014.01.009 24525225 PMC4028412

[65] Vaney MC, Rey FA. Class II enveloped viruses. Cell Microbiol. 13(10):1451–9. Epub 2011/07/28. 10.1111/j.1462-5822.2011.01653.x 21790946

[66] Kabsch W. Xds. Acta Crystallogr D Biol Crystallogr. 2010;66(Pt 2):125–32. Epub 2010/02/04. PubMed Central PMCID: PMC2815665. 10.1107/S0907444909047337 20124692 PMC2815665

[67] Adams PD, Grosse-Kunstleve RW, Hung LW, Ioerger TR, McCoy AJ, Moriarty NW, et al PHENIX: building new software for automated crystallographic structure determination. Acta Crystallogr D Biol Crystallogr. 2002;58(Pt 11):1948–54. Epub 2002/10/24. 12393927 10.1107/s0907444902016657

[68] Emsley P, Cowtan K. Coot: model-building tools for molecular graphics. Acta Crystallogr D Biol Crystallogr. 2004;60(Pt 12 Pt 1):2126–32. Epub 2004/12/02. 15572765 10.1107/S0907444904019158

[69] Murshudov GN, Vagin AA, Dodson EJ. Refinement of macromolecular structures by the maximum-likelihood method. Acta Crystallogr D Biol Crystallogr. 1997;53(Pt 3):240–55. 10.1107/S0907444996012255 15299926

[70] Pettersen EF, Goddard TD, Huang CC, Couch GS, Greenblatt DM, Meng EC, et al UCSF Chimera—a visualization system for exploratory research and analysis. J Comput Chem. 2004;25(13):1605–12. 10.1002/jcc.20084 15264254

[71] Baker NA, Sept D, Joseph S, Holst MJ, McCammon JA. Electrostatics of nanosystems: application to microtubules and the ribosome. Proceedings of the National Academy of Sciences of the United States of America. 2001;98(18):10037–41. PubMed Central PMCID: PMCPMC56910. 10.1073/pnas.181342398 11517324 PMC56910

[72] Collaborative Computational Project N. The CCP4 suite: programs for protein crystallography. Acta Crystallogr D Biol Crystallogr. 1994;50(Pt 5):760–3. 10.1107/S0907444994003112 15299374

[73] Glaser F, Pupko T, Paz I, Bell RE, Bechor-Shental D, Martz E, et al ConSurf: identification of functional regions in proteins by surface-mapping of phylogenetic information. Bioinformatics. 2003;19(1):163–4. 12499312 10.1093/bioinformatics/19.1.163

[74] Cifuentes-Munoz N, Darlix JL, Tischler ND. Development of a lentiviral vector system to study the role of the Andes virus glycoproteins. Virus Res. 2010;153(1):29–35. 10.1016/j.virusres.2010.07.001 20619306

[75] Brocato RL, Josleyn MJ, Wahl-Jensen V, Schmaljohn CS, Hooper JW. Construction and nonclinical testing of a Puumala virus synthetic M gene-based DNA vaccine. Clin Vaccine Immunol. 2013;20(2):218–26. PubMed Central PMCID: PMCPMC3571280. 10.1128/CVI.00546-12 23239797 PMC3571280

[76] Godoy P, Marsac D, Stefas E, Ferrer P, Tischler ND, Pino K, et al Andes virus antigens are shed in urine of patients with acute hantavirus cardiopulmonary syndrome. Journal of virology. 2009;83(10):5046–55. PubMed Central PMCID: PMC2682085. 10.1128/JVI.02409-08 19279096 PMC2682085

[77] Holm L, Rosenstrom P. Dali server: conservation mapping in 3D. Nucleic Acids Res. 2010;38(Web Server issue):W545–9. PubMed Central PMCID: PMCPMC2896194. 10.1093/nar/gkq366 20457744 PMC2896194

[78] Konagurthu AS, Whisstock JC, Stuckey PJ, Lesk AM. MUSTANG: a multiple structural alignment algorithm. Proteins. 2006;64(3):559–74. 10.1002/prot.20921 16736488

[79] Clamp M, Cuff J, Searle SM, Barton GJ. The Jalview Java alignment editor. Bioinformatics. 2004;20(3):426–7. 10.1093/bioinformatics/btg430 14960472

[80] Waterhouse AM, Procter JB, Martin DM, Clamp M, Barton GJ. Jalview Version 2—a multiple sequence alignment editor and analysis workbench. Bioinformatics. 2009;25(9):1189–91. PubMed Central PMCID: PMCPMC2672624. 10.1093/bioinformatics/btp033 19151095 PMC2672624

[81] Katoh K, Misawa K, Kuma K, Miyata T. MAFFT: a novel method for rapid multiple sequence alignment based on fast Fourier transform. Nucleic Acids Res. 2002;30(14):3059–66. PubMed Central PMCID: PMCPMC135756. 12136088 10.1093/nar/gkf436 PMC135756

