# Supplementary figures and images for: Crystal Structure of Glycoprotein C from a Hantavirus in the Post-fusion Conformation
Source: PLoS Pathog. 2016 Oct 26;12(10):e1005948. doi: 10.1371/journal.ppat.1005948 (PMC5081248; doi:10.1371/journal.ppat.1005948)

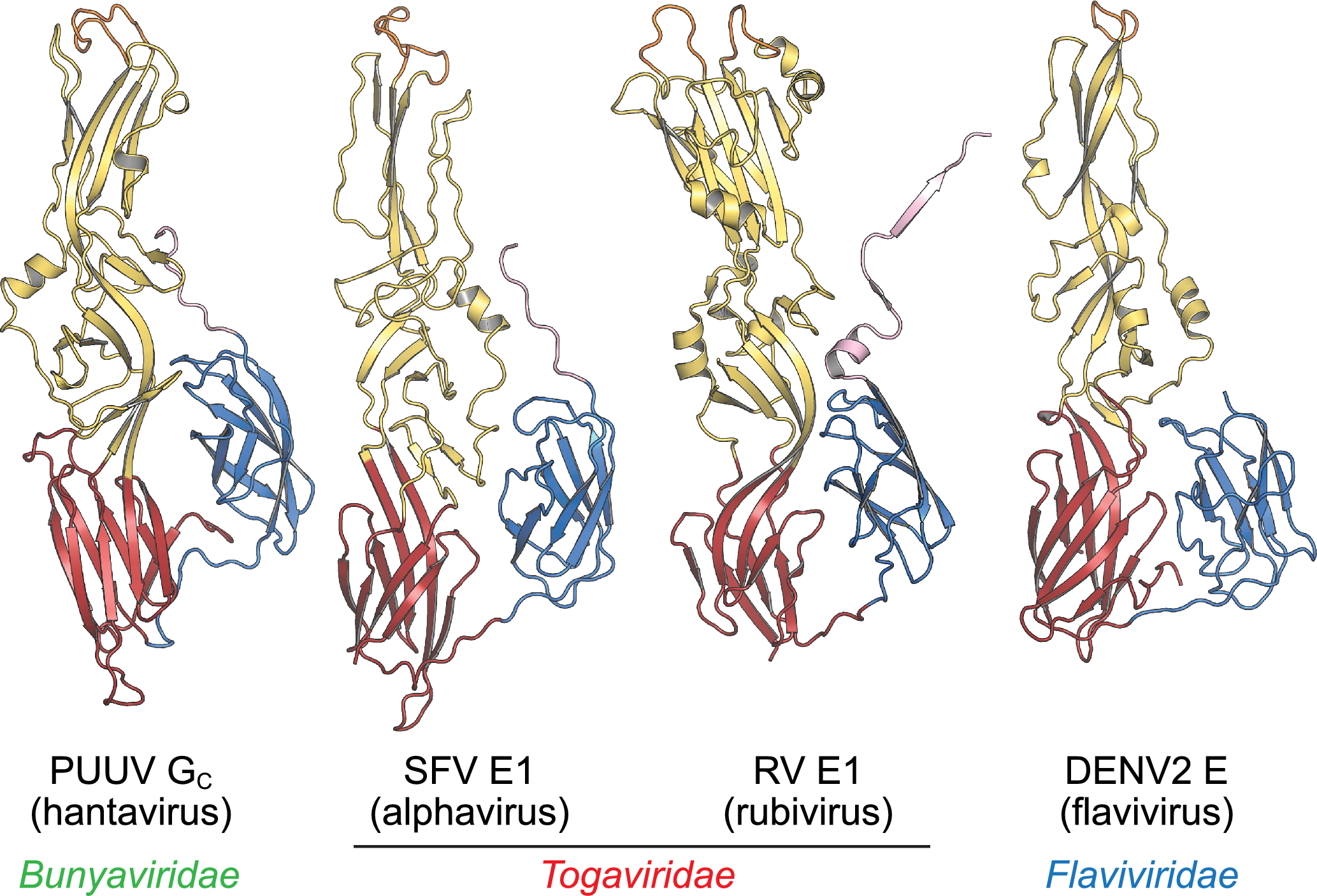

Supplement: S1 Fig — From left: crystal structures of PUUV sGC, Semliki forest virus E1 (PDB entry 1RER), Rubella virus E1 (PDB entry 4ADI) and Dengue virus glycoprotein E (PDB entry 1OK8) in their post-fusion conformation. To simplify, only one protomer from each trimer is shown. (TIF) [file ppat.1005948.s001.tif]

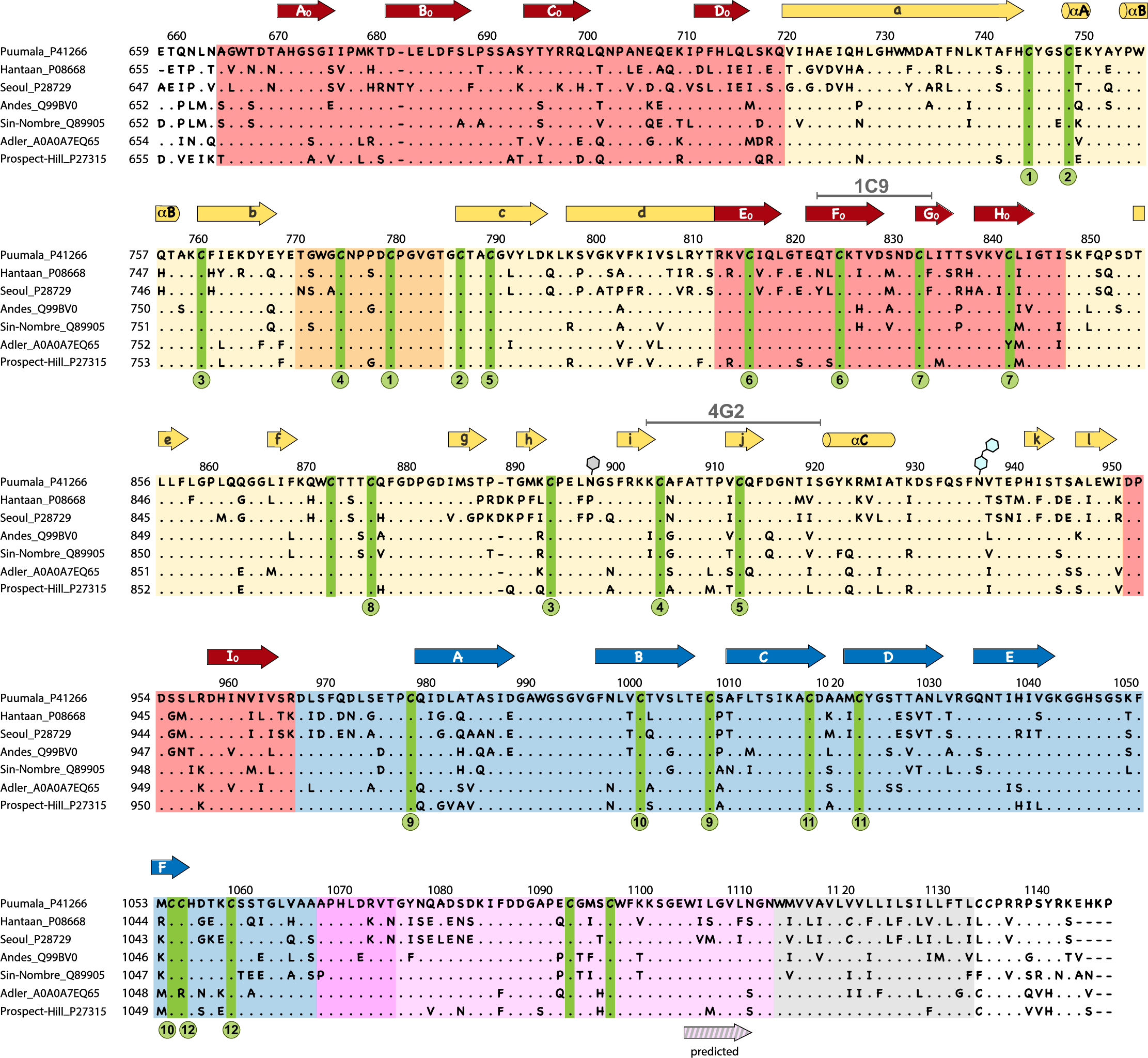

Supplement: S2 Fig — Amino acid sequence alignment of GC from selected hantaviruses. Conserved residues were replaced with periods. Domain colors are as in Fig 1. Arrows denote β-strands and cylinders represent α-helices. Glycans are represented by cyan hexagons and disulfide bonds are indicated in green. The unoccupied glycosylation site is represented with single grey hexagon. Light pink and gray shading regions corresponds the unmodeled stem and transmembrane regions, respectively. Cytoplasmic C-terminal tail presented with no shading. Secondary structure prediction of the C-terminal β-strand is represented by a pink/grey arrow. Numbered green circles represent cysteine residues, where cysteine residues with the same numbering are disulfide linked. Black bars indicate neutralizing epitopes. Database sequence accession codes are per legend and correspond to the Uniprot database (http://www.uniprot.org). (TIF) [file ppat.1005948.s002.tif]

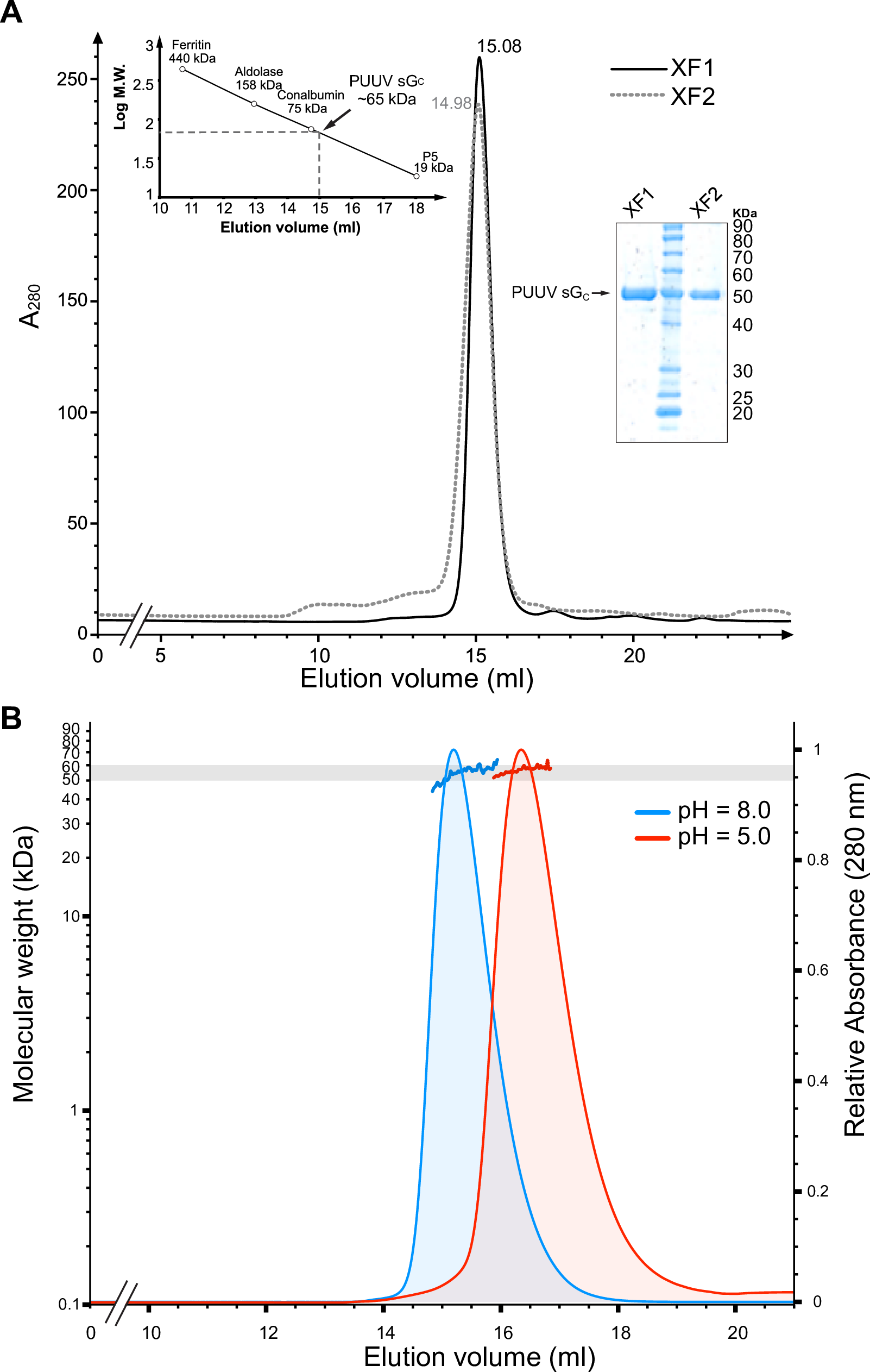

Supplement: S3 Fig — (A) A total of 0.2 mL of sGC (1 g/L) was loaded onto a Superdex 200 (30/100) size-exclusion column pre-equilibrated with 20 mM Tris buffer pH 8.0 and 100 mM NaCl. The eluate was analyzed for absorbance at 280 nm. Inset: Standard curve obtained with proteins of known masses. The position of GC on the curve is indicated with an arrow. The corresponding MW of sGC was calculated using the line equations of a standard curve. The MW of sGC calculated from the sequence is 49.3 KDa excluding glycosylations. On the right, a Coomassie stained SDS-PAGE analysis of the two preparations. (B) SEC-MALS analysis of PUUV GC in different pHs. 0.2 mL at 2.5 g/L were loaded onto Superdex 200 column at pH 8.0 and pH 5.0. The elution was analyzed for absorbance at 280 nm (right y axis) and for multiangle light scattering, which was converted to molecular mass (Left y axis; material and methods). Gray rectangle represents the Mw range between 50–60 KDa. Colors are as per legend. (TIF) [file ppat.1005948.s003.tif]

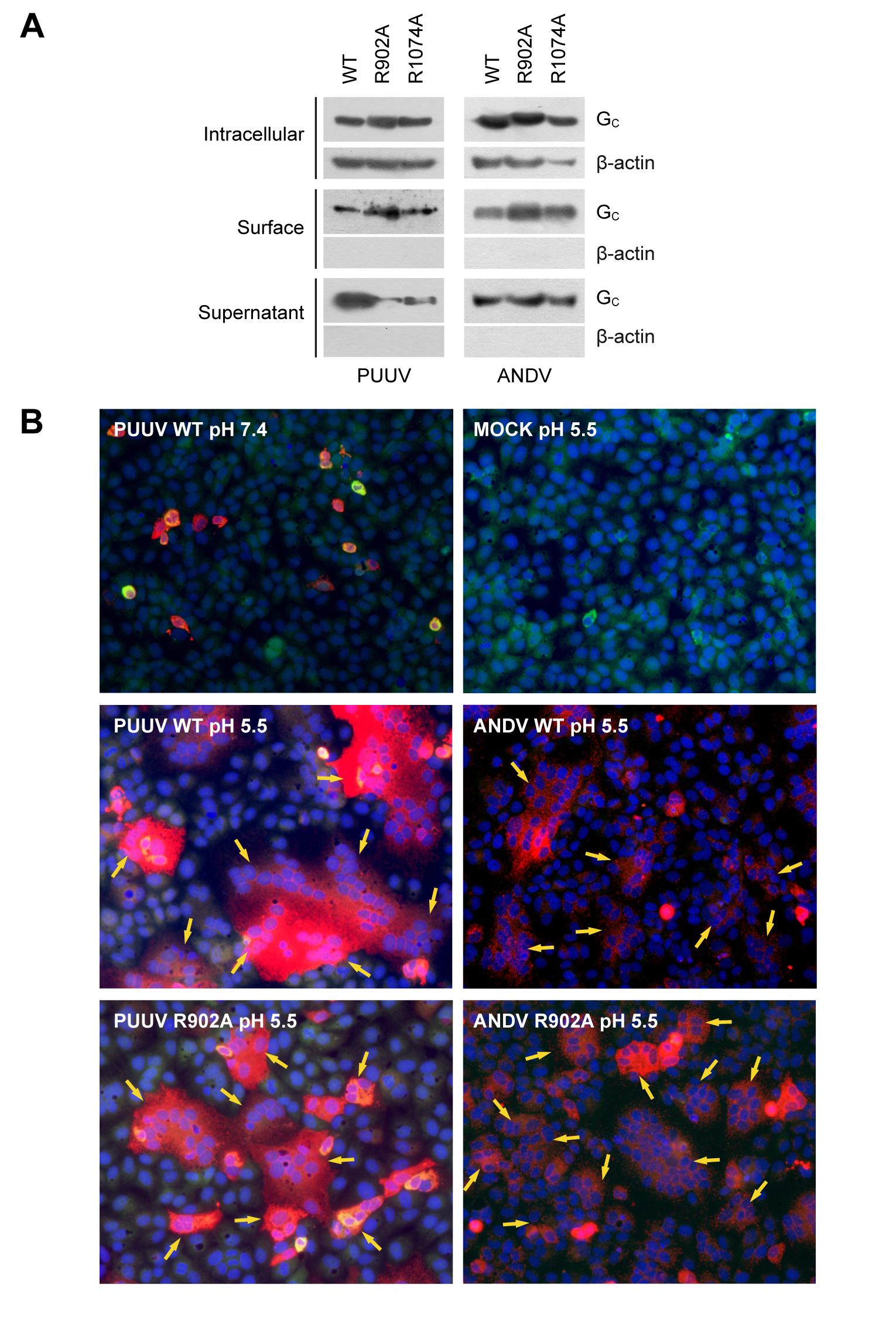

Supplement: S4 Fig — (A) Western blot analysis of the presence of GC in different cellular fractions and the supernatant of 293 FT cells expressing wild type or mutant GPC from PUUV and ANDV. Fractions correspond to non-biotinylated intracellular fraction, biotinylated cell surface fraction and the concentrated supernatant of cells. (B) Representative fluorescence micrographs of Vero E6 cells expressing wild type or R902A mutant GPC from PUUV or ANDV, and treated at different pHs. The cell cytoplasm was labelled with 5-chloromethylfluorescein diacetate (CMFDA; green fluorescence), nuclei with DAPI (blue fluorescence) and GC was detected with anti-GC MAb (Alexa555; red fluorescence). Cells from a partial microscopy field are shown from a representative experiment. Mock indicates cells transfected with an empty expression plasmid. Arrows indicate syncytia. (200 X magnification). Quantitative analysis of these cell-cell fusion assays is presented also in Fig 6C. (TIF) [file ppat.1005948.s004.tif]

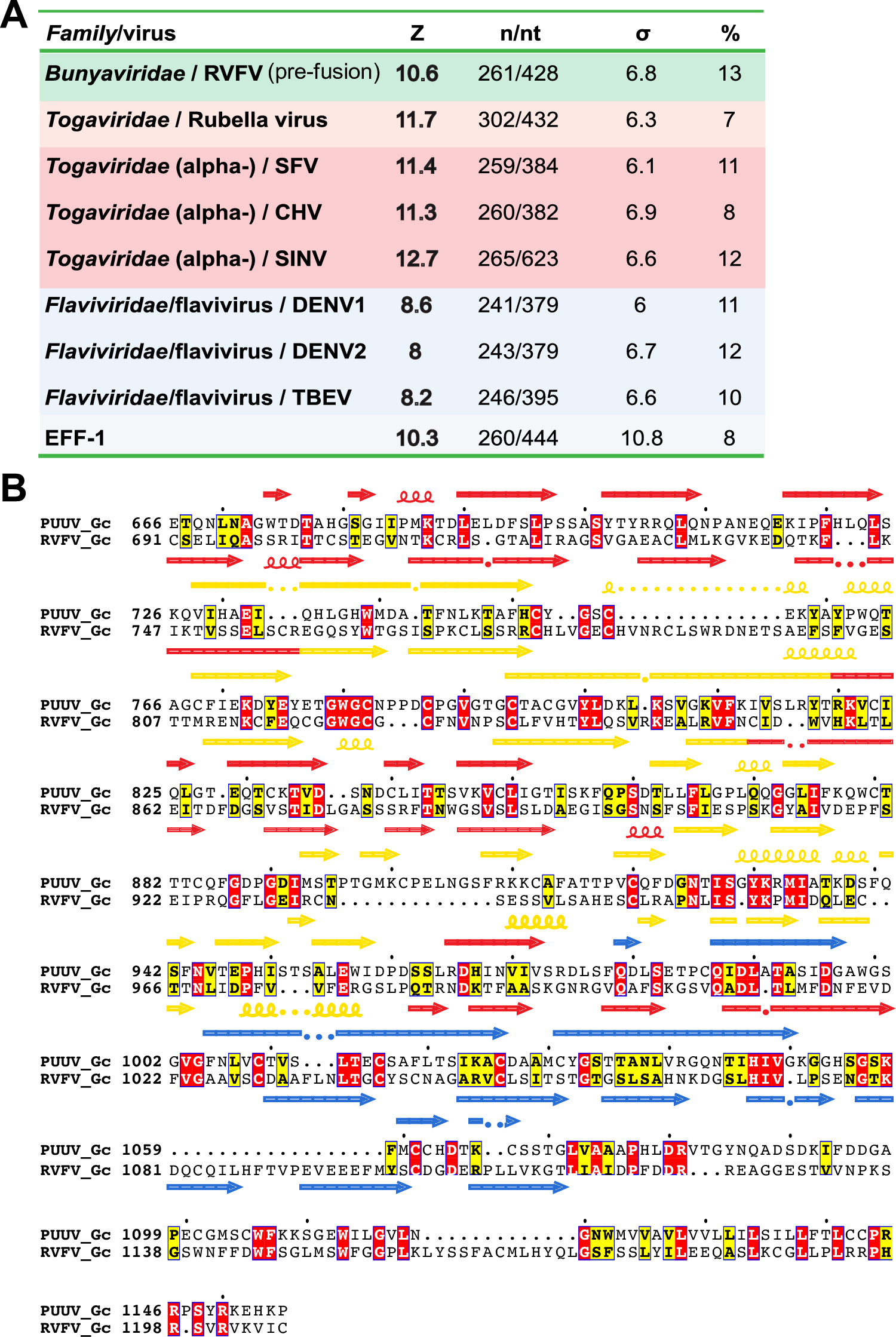

Supplement: S5 Fig — Structural alignment and comparison of PUUV with other class II membrane fusion proteins (A) PUUV GC shows more structural similarity to alphaviruses then to other class II proteins. Table represents the DALI server (http://ekhidna.biocenter.helsinki.fi/dali_server/start) scores with PUUV GC as the query. Z-score describe the statistical significance of a pairwise comparison score (higher score represents higher similarity), n/nt is the ratio between the number of aligned residues (n) and total residues in the structure (nt), σ is the Root mean square deviation (RMSD) for the aligned residues and % represents sequence identity. Bunyaviridae are in greens, Togaviridae in reds, Flaviviridae in blues and eukaryotes are in grey. (B) Sequence alignment of PUUV and RVFV GC proteins. Alignment was obtained using MAFFT [81] the secondary structure assignment for RVFV was based on PDB entry 4HJ1. Colors scheme is as in Fig 1. (TIF) [file ppat.1005948.s005.tif]
